# Supplementary material for: Generalized probabilistic canonical correlation analysis for multi-modal data integration with full or partial observations
Source: BMC Bioinformatics. 2025 Oct 16;26:249. doi: 10.1186/s12859-025-06227-9 (PMC12533326; doi:10.1186/s12859-025-06227-9)
Supplement: Supplementary file 1 — (pdf 13978 KB) [file 12859_2025_6227_MOESM1_ESM.pdf]

Supplementary File for "Generalized Probabilistic Canonical Correlation Analysis for Multi-modal Data Integration with Full or Partial Observations"

1 Supplementary Methods

1.1 GPCCA Model and Its Estimation Framework

1.1.1 Model Specification

In the Methods section, we have constructed the GPCCA model as follows:

$$\underset{m\times n}{\mathbf{X}} = \underset{m\times d}{\mathbf{W}} \cdot \underset{d\times n}{\mathbf{Z}} + \underset{m\times n}{\mathbf{U}} + \underset{m\times n}{\mathbf{E}},$$

where $m = \sum_{r=1}^{R} m_r$ and $1 \leq d \leq \min\{m_r\}_{1\leq r\leq R}$. In addition,

$$\{\mathbf{z}_k\}_{1\leq k\leq n} \overset{\text{i.i.d.}}{\sim} \mathcal{N}_d(\mathbf{0}, \mathbf{I}) \text{ and } \{\boldsymbol{\epsilon}_k\}_{1\leq k\leq n} \overset{\text{i.i.d.}}{\sim} \mathcal{N}_m(\mathbf{0}, \boldsymbol{\Psi}) \text{ where } \boldsymbol{\Psi} = \begin{pmatrix} \boldsymbol{\Psi}^{(1)} & & & \\ & \boldsymbol{\Psi}^{(2)} & & \\ & & \ddots & \\ & & & \boldsymbol{\Psi}^{(R)} \end{pmatrix}.$$

The error covariance matrix $\boldsymbol{\Psi}$ is block diagonal, allowing for potential correlations between error terms within the same modality, while assuming no correlations between error terms across modalities. This structure represents a trade-off between the overly simplistic diagonal covariance matrix and the fully parameterized covariance matrix, which may lead to overfitting and robustness issues.

1.1.2 Expectation of Complete Log-likelihood

Denote the model parameter set as $\boldsymbol{\Theta} = (\mathbf{W}, \boldsymbol{\mu}, \boldsymbol{\Psi})$, then the complete likelihood is:

$$\mathcal{L}_{\mathsf{c}}(\boldsymbol{\Theta}; \mathbf{X}, \mathbf{Z}) = \mathcal{P}(\mathbf{X}, \mathbf{Z}; \boldsymbol{\Theta}) = \mathcal{P}(\mathbf{X}|\mathbf{Z}; \boldsymbol{\Theta})\mathcal{P}(\mathbf{Z}; \boldsymbol{\Theta}) := \mathcal{P}(\mathbf{X}|\mathbf{Z})\mathcal{P}(\mathbf{Z}).$$

It follows that the complete log-likelihood is:

$$\begin{aligned}\ell_{\mathsf{c}}(\boldsymbol{\Theta}) &:= \ell_{\mathsf{c}}(\boldsymbol{\Theta} : \mathbf{X}, \mathbf{Z}) = \ln \mathcal{L}_{\mathsf{c}}(\boldsymbol{\Theta} : \mathbf{X}, \mathbf{Z}) = \ln \mathcal{P}(\mathbf{X}|\mathbf{Z}) + \ln \mathcal{P}(\mathbf{Z}) \\ &= \ln \prod_{k=1}^{n} p(\mathbf{x}_k|\mathbf{z}_k) + \ln \prod_{k=1}^{n} p(\mathbf{z}_k) = \sum_{k=1}^{n} \ln p(\mathbf{x}_k|\mathbf{z}_k) + \sum_{k=1}^{n} \ln p(\mathbf{z}_k)\end{aligned}$$

$$= \sum_{k=1}^{n} \ln(2\pi)^{-\frac{m}{2}} |\mathbf{\Psi}|^{-\frac{1}{2}} \exp(-\frac{1}{2}(\mathbf{x}_k - \boldsymbol{\mu} - \mathbf{W}\mathbf{z}_k)^\mathsf{T} \mathbf{\Psi}^{-1} (\mathbf{x}_k - \boldsymbol{\mu} - \mathbf{W}\mathbf{z}_k)) + \sum_{k=1}^{n} \ln(2\pi)^{-\frac{d}{2}} \exp(-\frac{1}{2}\mathbf{z}_k^\mathsf{T}\mathbf{z}_k)$$

$$= -n\frac{m}{2}\ln 2\pi - \frac{n}{2}\ln|\mathbf{\Psi}| - \frac{1}{2}\sum_{k=1}^{n}(\mathbf{x}_k - \boldsymbol{\mu} - \mathbf{W}\mathbf{z}_k)^\mathsf{T} \mathbf{\Psi}^{-1}(\mathbf{x}_k - \boldsymbol{\mu} - \mathbf{W}\mathbf{z}_k) - n\frac{d}{2}\ln 2\pi - \frac{1}{2}\sum_{k=1}^{n}\mathbf{z}_k^\mathsf{T}\mathbf{z}_k$$

$$\propto -\frac{n}{2}\ln|\mathbf{\Psi}| - \frac{1}{2}\sum_{k=1}^{n}\mathsf{tr}(\mathbf{\Psi}^{-1}(\mathbf{x}_k - \boldsymbol{\mu} - \mathbf{W}\mathbf{z}_k)(\mathbf{x}_k - \boldsymbol{\mu} - \mathbf{W}\mathbf{z}_k)^\mathsf{T}) - \frac{1}{2}\sum_{k=1}^{n}\mathsf{tr}(\mathbf{z}_k\mathbf{z}_k^\mathsf{T})$$

$$= -\frac{n}{2}\ln|\mathbf{\Psi}| - \frac{1}{2}\sum_{k=1}^{n}\mathsf{tr}(\mathbf{z}_k\mathbf{z}_k^\mathsf{T})$$

$$-\frac{1}{2}\sum_{k=1}^{n}\mathsf{tr}(\mathbf{\Psi}^{-1}(\mathbf{x}_k\mathbf{x}_k^\mathsf{T} + \boldsymbol{\mu}\boldsymbol{\mu}^\mathsf{T} + \mathbf{W}\mathbf{z}_k\mathbf{z}_k^\mathsf{T}\mathbf{W}^\mathsf{T} - 2\mathbf{x}_k\boldsymbol{\mu}^\mathsf{T} + 2\mathbf{W}\mathbf{z}_k\boldsymbol{\mu}^\mathsf{T} - 2\mathbf{W}\mathbf{z}_k\mathbf{x}_k^\mathsf{T})).$$

Then, we define the Q-function as

$$\mathcal{Q}(\mathbf{\Theta}) = -\frac{n}{2}\ln|\mathbf{\Psi}| - \frac{1}{2}\sum_{k=1}^{n}\mathsf{tr}(\mathbf{\Psi}^{-1}\mathbf{G}_k) - \frac{1}{2}\sum_{k=1}^{n}\mathsf{tr}(\mathbb{E}(\mathbf{z}_k\mathbf{z}_k^\mathsf{T})),$$

where $\mathbf{G}_k = \mathbb{E}(\mathbf{x}_k\mathbf{x}_k^\mathsf{T}) + \boldsymbol{\mu}\boldsymbol{\mu}^\mathsf{T} + \mathbf{W}\,\mathbb{E}(\mathbf{z}_k\mathbf{z}_k^\mathsf{T})\mathbf{W}^\mathsf{T} - 2\,\mathbb{E}(\mathbf{x}_k)\boldsymbol{\mu}^\mathsf{T} + 2\mathbf{W}\,\mathbb{E}(\mathbf{z}_k)\boldsymbol{\mu}^\mathsf{T} - 2\mathbf{W}\,\mathbb{E}(\mathbf{z}_k\mathbf{x}_k^\mathsf{T})$. Therefore, we have $\mathbb{E}[\ell_\mathsf{c}(\mathbf{\Theta})] \propto \mathcal{Q}(\mathbf{\Theta})$.

1.1.3 Gradients and Stationary Points

To maximize $\mathcal{Q}(\mathbf{\Theta})$, we need to derive its gradient and find the stationary point of $\dfrac{\partial\mathcal{Q}(\mathbf{\Theta})}{\partial\mathbf{\Theta}} = \mathbf{0}$.

Mean Vector $\boldsymbol{\mu}$

Taking partial derivative of $\mathcal{Q}(\mathbf{\Theta})$ w.r.t. mean vector $\boldsymbol{\mu}$ gives the following

$$\begin{aligned}
\frac{\partial\mathcal{Q}(\mathbf{\Theta})}{\partial\boldsymbol{\mu}} &= -\frac{1}{2}\sum_{k=1}^{n}\frac{\partial}{\partial\boldsymbol{\mu}}\mathsf{tr}(\mathbf{\Psi}^{-1}\mathbf{G}_k) \\
&= -\frac{1}{2}\sum_{k=1}^{n}\frac{\partial}{\partial\boldsymbol{\mu}}\mathsf{tr}(\mathbf{\Psi}^{-1}\boldsymbol{\mu}\boldsymbol{\mu}^\mathsf{T} - 2\mathbf{\Psi}^{-1}\,\mathbb{E}(\mathbf{x}_k)\boldsymbol{\mu}^\mathsf{T} + 2\mathbf{\Psi}^{-1}\mathbf{W}\,\mathbb{E}(\mathbf{z}_k)\boldsymbol{\mu}^\mathsf{T}) \\
&= -\frac{1}{2}\sum_{k=1}^{n}(\frac{\partial\mathsf{tr}(\boldsymbol{\mu}^\mathsf{T}\mathbf{\Psi}^{-1}\boldsymbol{\mu})}{\partial\boldsymbol{\mu}} - 2\frac{\partial\mathsf{tr}(\boldsymbol{\mu}^\mathsf{T}\mathbf{\Psi}^{-1}\,\mathbb{E}(\mathbf{x}_k))}{\partial\boldsymbol{\mu}} + 2\frac{\partial\mathsf{tr}(\boldsymbol{\mu}^\mathsf{T}\mathbf{\Psi}^{-1}\mathbf{W}\,\mathbb{E}(\mathbf{z}_k))}{\partial\boldsymbol{\mu}}) \\
&= -\frac{1}{2}\sum_{k=1}^{n}(\frac{\partial(\boldsymbol{\mu}^\mathsf{T}\mathbf{\Psi}^{-1}\boldsymbol{\mu})}{\partial\boldsymbol{\mu}} - 2\frac{\partial(\boldsymbol{\mu}^\mathsf{T}\mathbf{\Psi}^{-1}\,\mathbb{E}(\mathbf{x}_k))}{\partial\boldsymbol{\mu}} + 2\frac{\partial(\boldsymbol{\mu}^\mathsf{T}\mathbf{\Psi}^{-1}\mathbf{W}\,\mathbb{E}(\mathbf{z}_k))}{\partial\boldsymbol{\mu}}) \\
&= -\frac{1}{2}\sum_{k=1}^{n}(2\mathbf{\Psi}^{-1}\boldsymbol{\mu} - 2\mathbf{\Psi}^{-1}\,\mathbb{E}(\mathbf{x}_k) + 2\mathbf{\Psi}^{-1}\mathbf{W}\,\mathbb{E}(\mathbf{z}_k)).
\end{aligned}$$

Setting $\dfrac{\partial\mathcal{Q}(\mathbf{\Theta})}{\partial\boldsymbol{\mu}} = \mathbf{0}$ gives

$$\hat{\boldsymbol{\mu}} = \frac{1}{n}\sum_{k=1}^{n}(\mathbb{E}(\mathbf{x}_k) - \mathbf{W}\,\mathbb{E}(\mathbf{z}_k)).$$

Loading Matrix $\mathbf{W}$

Taking partial derivative of $\mathcal{Q}(\mathbf{\Theta})$ w.r.t. loading matrix $\mathbf{W}$ gives the following

$$\begin{aligned}\frac{\partial \mathcal{Q}(\mathbf{\Theta})}{\partial \mathbf{W}} &= -\frac{1}{2}\sum_{k=1}^{n}\frac{\partial}{\partial \mathbf{W}}\text{tr}(\mathbf{\Psi}^{-1}\mathbf{G}_k)\\ &= -\frac{1}{2}\sum_{k=1}^{n}\frac{\partial}{\partial \mathbf{W}}\text{tr}(\mathbf{\Psi}^{-1}\mathbf{W}\,\mathbb{E}(\mathbf{z}_k\mathbf{z}_k^{\mathsf{T}})\mathbf{W}^{\mathsf{T}} + 2\mathbf{\Psi}^{-1}\mathbf{W}\,\mathbb{E}(\mathbf{z}_k)\boldsymbol{\mu}^{\mathsf{T}} - 2\mathbf{\Psi}^{-1}\mathbf{W}\,\mathbb{E}(\mathbf{z}_k\mathbf{x}_k^{\mathsf{T}}))\\ &= -\frac{1}{2}\sum_{k=1}^{n}(\frac{\partial\text{tr}(\mathbf{\Psi}^{-1}\mathbf{W}\,\mathbb{E}(\mathbf{z}_k\mathbf{z}_k^{\mathsf{T}})\mathbf{W}^{\mathsf{T}})}{\partial \mathbf{W}} + 2\frac{\partial\text{tr}(\mathbf{\Psi}^{-1}\mathbf{W}\,\mathbb{E}(\mathbf{z}_k)\boldsymbol{\mu}^{\mathsf{T}})}{\partial \mathbf{W}} - 2\frac{\partial\text{tr}(\mathbf{\Psi}^{-1}\mathbf{W}\,\mathbb{E}(\mathbf{z}_k\mathbf{x}_k^{\mathsf{T}}))}{\partial \mathbf{W}})\\ &= -\frac{1}{2}\sum_{k=1}^{n}(2\mathbf{\Psi}^{-1}\mathbf{W}\,\mathbb{E}(\mathbf{z}_k\mathbf{z}_k^{\mathsf{T}}) + 2\mathbf{\Psi}^{-1}\boldsymbol{\mu}(\mathbb{E}(\mathbf{z}_k))^{\mathsf{T}} - 2\mathbf{\Psi}^{-1}\,\mathbb{E}(\mathbf{x}_k\mathbf{z}_k^{\mathsf{T}})).\end{aligned}$$

Setting $\dfrac{\partial \mathcal{Q}(\mathbf{\Theta})}{\partial \mathbf{W}} = 0$ gives

$$\hat{\mathbf{W}} = [\sum_{k=1}^{n}(\mathbb{E}(\mathbf{x}_k\mathbf{z}_k^{\mathsf{T}}) - \boldsymbol{\mu}(\mathbb{E}(\mathbf{z}_k))^{\mathsf{T}})][\sum_{k=1}^{n}\mathbb{E}(\mathbf{z}_k\mathbf{z}_k^{\mathsf{T}})]^{-1}.$$

Error Covariance Matrix $\mathbf{\Psi}$

Taking partial derivative of $\mathcal{Q}(\mathbf{\Theta})$ w.r.t. error covariance matrix $\mathbf{\Psi}$ gives the following

$$\frac{\partial \mathcal{Q}(\mathbf{\Theta})}{\partial \mathbf{\Psi}} = -\frac{n}{2}\frac{\partial}{\partial \mathbf{\Psi}}\ln|\mathbf{\Psi}| - \frac{1}{2}\sum_{k=1}^{n}\frac{\partial}{\partial \mathbf{\Psi}}\text{tr}(\mathbf{\Psi}^{-1}\mathbf{G}_k) = -\frac{n}{2}\mathbf{\Psi}^{-1} + \frac{1}{2}\sum_{k=1}^{n}\mathbf{\Psi}^{-1}\mathbf{G}_k^{\mathsf{T}}\mathbf{\Psi}^{-1}.$$

Setting $\dfrac{\partial \mathcal{Q}(\mathbf{\Theta})}{\partial \mathbf{\Psi}} = \mathbf{0}$ gives

$$\hat{\mathbf{\Psi}} = \frac{1}{n}\sum_{k=1}^{n}\mathbf{G}_k^{\mathsf{T}}.$$

Due to the block diagonal structure of $\mathbf{\Psi}$, we only retain its block diagonal elements $\hat{\mathbf{\Psi}} = \frac{1}{n}\text{Bdiag}_{\mathbf{\Psi}}[\sum_{k=1}^{n}\mathbf{G}_k^{\mathsf{T}}] = \frac{1}{n}\sum_{k=1}^{n}\text{Bdiag}_{\mathbf{\Psi}}[\mathbf{G}_k^{\mathsf{T}}]$. Here, $\text{Bdiag}_{\mathbf{\Psi}}(\cdot)$ is a function that only retains the corresponding elements of its input matrix w.r.t. the block diagonal structure of $\mathbf{\Psi}$ (resulting in the same structure as $\mathbf{\Psi}$). In fact, $\hat{\mathbf{\Psi}} = \frac{1}{n}\sum_{k=1}^{n}\text{Bdiag}_{\mathbf{\Psi}}[\mathbf{G}_k^{\mathsf{T}}]$ can be naturally obtained by setting $\dfrac{\partial \mathcal{Q}(\mathbf{\Theta})}{\partial \mathbf{\Psi}^{(r)}} = \mathbf{0}$ for each modality. A proof of equivalence is provided below.

Proof:

$$\mathbf{\Psi} = \text{diag}(\mathbf{\Psi}^{(1)}, \mathbf{\Psi}^{(2)}, \ldots, \mathbf{\Psi}^{(R)}) \Rightarrow \mathbf{\Psi}^{-1} = \text{diag}({\mathbf{\Psi}^{(1)}}^{-1}, {\mathbf{\Psi}^{(2)}}^{-1}, \ldots, {\mathbf{\Psi}^{(R)}}^{-1})$$

Suppose $\text{Bdiag}_{\mathbf{\Psi}}[\mathbf{G}_k] = \text{diag}(\mathbf{G}_k^{(1)}, \mathbf{G}_k^{(2)}, \ldots, \mathbf{G}_k^{(R)})$, and since operator $\text{tr}(\cdot)$ only applies to the main diagonal elements of its input matrix, then we have

$$\text{tr}(\mathbf{\Psi}^{-1}\mathbf{G}_k) = \text{tr}(\text{diag}({\mathbf{\Psi}^{(1)}}^{-1}\mathbf{G}_k^{(1)}, {\mathbf{\Psi}^{(2)}}^{-1}\mathbf{G}_k^{(2)}, \ldots, {\mathbf{\Psi}^{(R)}}^{-1}\mathbf{G}_k^{(R)})) = \sum_{r=1}^{R}\text{tr}({\mathbf{\Psi}^{(r)}}^{-1}\mathbf{G}_k^{(r)}).$$

Taking partial derivative of $\mathcal{Q}(\mathbf{\Theta})$ w.r.t. $\mathbf{\Psi}^{(r)}$ on the r-th modality gives the following

$$\begin{aligned}\frac{\partial \mathcal{Q}(\mathbf{\Theta})}{\partial \mathbf{\Psi}^{(r)}} &= -\frac{n}{2}\frac{\partial}{\partial \mathbf{\Psi}^{(r)}}\ln \prod_{r=1}^{R}|\mathbf{\Psi}^{(r)}| - \frac{1}{2}\sum_{k=1}^{n}\frac{\partial}{\partial \mathbf{\Psi}^{(r)}}\mathsf{tr}(\mathbf{\Psi}^{-1}\mathbf{G}_k) \\ &= -\frac{n}{2}\frac{\partial}{\partial \mathbf{\Psi}^{(r)}}\sum_{r=1}^{R}\ln|\mathbf{\Psi}^{(r)}| - \frac{1}{2}\sum_{k=1}^{n}\frac{\partial}{\partial \mathbf{\Psi}^{(r)}}\sum_{r=1}^{R}\mathsf{tr}(\mathbf{\Psi}^{(r)^{-1}}\mathbf{G}_k^{(r)}) \\ &= -\frac{n}{2}\mathbf{\Psi}^{(r)^{-1}} + \frac{1}{2}\sum_{k=1}^{n}\mathbf{\Psi}^{(r)^{-1}}\mathbf{G}_k^{(r)^{\mathsf{T}}}\mathbf{\Psi}^{(r)^{-1}}.\end{aligned}$$

Similarly, setting $\frac{\partial \mathcal{Q}(\mathbf{\Theta})}{\partial \mathbf{\Psi}^{(r)}} = \mathbf{0}$ modal-wisely gives the MLE of $\mathbf{\Psi}^{(r)}$: $\hat{\mathbf{\Psi}}^{(r)} = \frac{1}{n}\sum_{k=1}^{n}\mathbf{G}_k^{(r)^{\mathsf{T}}}$, then

$$\hat{\mathbf{\Psi}} = \mathsf{diag}(\hat{\mathbf{\Psi}}^{(1)}, \hat{\mathbf{\Psi}}^{(2)}, \ldots, \hat{\mathbf{\Psi}}^{(R)}) = \frac{1}{n}\sum_{k=1}^{n}\mathsf{diag}(\mathbf{G}_k^{(1)^{\mathsf{T}}}, \mathbf{G}_k^{(2)^{\mathsf{T}}}, \ldots, \mathbf{G}_k^{(R)^{\mathsf{T}}}) = \frac{1}{n}\sum_{k=1}^{n}\mathsf{Bdiag}_{\mathbf{\Psi}}[\mathbf{G}_k^{\mathsf{T}}].$$

1.1.4 Conditional Expectations

With missing values and the key components defined for the t-th iteration of EM algorithm in the Methods section, the generative structure stays unchanged for the observed data.

$$\tilde{\mathbf{x}}_k|\mathbf{z}_k \sim \mathcal{N}(\tilde{\mathbf{W}}_k^{(t)}\mathbf{z}_k + \tilde{\boldsymbol{\mu}}_k^{(t)}, \tilde{\mathbf{\Psi}}_k^{(t)}) \Rightarrow \begin{bmatrix}\mathbf{z}_k \\ \tilde{\mathbf{x}}_k\end{bmatrix} \sim \mathcal{N}(\begin{bmatrix}\mathbf{0} \\ \tilde{\boldsymbol{\mu}}_k^{(t)}\end{bmatrix}, \begin{bmatrix}\mathbf{I} & \tilde{\mathbf{W}}_k^{(t)\mathsf{T}} \\ \tilde{\mathbf{W}}_k^{(t)} & \tilde{\mathbf{W}}_k^{(t)}\tilde{\mathbf{W}}_k^{(t)\mathsf{T}} + \tilde{\mathbf{\Psi}}_k^{(t)}\end{bmatrix})$$

Hence, the conditional distribution of $\mathbf{z}_k$ given observed data $\tilde{\mathbf{x}}_k$ is

$$\mathbf{z}_k|\tilde{\mathbf{x}}_k \sim \mathcal{N}(\tilde{\mathbf{W}}_k^{(t)\mathsf{T}}(\tilde{\mathbf{W}}_k^{(t)}\tilde{\mathbf{W}}_k^{(t)\mathsf{T}} + \tilde{\mathbf{\Psi}}_k^{(t)})^{-1}(\tilde{\mathbf{x}}_k - \tilde{\boldsymbol{\mu}}_k^{(t)}), \mathbf{I} - \tilde{\mathbf{W}}_k^{(t)\mathsf{T}}(\tilde{\mathbf{W}}_k^{(t)}\tilde{\mathbf{W}}_k^{(t)\mathsf{T}} + \tilde{\mathbf{\Psi}}_k^{(t)})^{-1}\tilde{\mathbf{W}}_k^{(t)}).$$

Find $\mathbb{E}(\mathbf{z}_k)$ and $\mathbb{E}(\mathbf{z}_k\mathbf{z}_k^{\mathsf{T}})$ given Observed Data:
Write $\tilde{\mathbf{M}}_k^{(t)} = (\mathbf{I} + \tilde{\mathbf{W}}_k^{(t)\mathsf{T}}[\tilde{\mathbf{\Psi}}_k^{(t)}]^{-1}\tilde{\mathbf{W}}_k^{(t)})^{-1}$, then with Woodbury matrix identity [1], it can be easily proved that $\mathbf{z}_k|\tilde{\mathbf{x}}_k \sim \mathcal{N}(\tilde{\mathbf{M}}_k^{(t)}\tilde{\mathbf{W}}_k^{(t)\mathsf{T}}[\tilde{\mathbf{\Psi}}_k^{(t)}]^{-1}(\tilde{\mathbf{x}}_k - \tilde{\boldsymbol{\mu}}_k^{(t)}), \tilde{\mathbf{M}}_k^{(t)})$. It follows that:

$$\begin{aligned}\mathbb{E}(\mathbf{z}_k) &:= \mathbb{E}(\mathbf{z}_k|\tilde{\mathbf{x}}_k; \Theta^{(t)}) = \tilde{\mathbf{M}}_k^{(t)}\tilde{\mathbf{W}}_k^{(t)\mathsf{T}}[\tilde{\mathbf{\Psi}}_k^{(t)}]^{-1}(\tilde{\mathbf{x}}_k - \tilde{\boldsymbol{\mu}}_k), \\ \mathbb{E}(\mathbf{z}_k\mathbf{z}_k^{\mathsf{T}}) &:= \mathbb{E}(\mathbf{z}_k\mathbf{z}_k^{\mathsf{T}}|\tilde{\mathbf{x}}_k; \Theta^{(t)}) = \mathsf{Cov}(\mathbf{z}_k, \mathbf{z}_k|\tilde{\mathbf{x}}_k) + \mathbb{E}(\mathbf{z}_k|\tilde{\mathbf{x}}_k)(\mathbb{E}(\mathbf{z}_k|\tilde{\mathbf{x}}_k))^{\mathsf{T}} = \tilde{\mathbf{M}}_k^{(t)} + \mathbb{E}(\mathbf{z}_k)(\mathbb{E}(\mathbf{z}_k))^{\mathsf{T}}.\end{aligned}$$

It is worth noting that using the Woodbury matrix identity allows us to invert the covariance matrix of our data $\mathbf{x}_k$ (or $\tilde{\mathbf{x}}_k$) in $O(d^3)$ time [1] rather than (at most) $O(m^3)$ time with Gauss-Jordan Elimination, where d and m are the dimensions of latent factor and data vector respectively.

Find $\mathbb{E}(\mathbf{x}_k)$ given Observed Data:
Here, we derive $\mathbb{E}(\mathbf{x}_k)$ element-wisely. Recall $\mathbf{x}_k := \mathbf{X}_{\cdot k}$, and for the i-th dimension of $\mathbf{x}_k$: $\mathbf{X}_{ik}$,

- if $\mathbf{O}_{ik} = 1$, then $\mathbb{E}(\mathbf{X}_{ik}) := \mathbb{E}(\mathbf{X}_{ik}|\tilde{\mathbf{x}}_k; \Theta^{(t)}) = \mathbf{X}_{ik}$
- if $\mathbf{O}_{ik} = 0$, then $\mathbb{E}(\mathbf{X}_{ik}) := \mathbb{E}(\mathbf{X}_{ik}|\tilde{\mathbf{x}}_k; \Theta^{(t)}) = \mathbb{E}(\mathbf{W}_{i\cdot}^{(t)}\mathbf{z}_k + \boldsymbol{\mu}_i^{(t)} + \mathbf{E}_{ik}|\tilde{\mathbf{x}}_k)$
$= \mathbf{W}_{i\cdot}^{(t)}\,\mathbb{E}(\mathbf{z}_k|\tilde{\mathbf{x}}_k) + \boldsymbol{\mu}_i^{(t)} + \mathbb{E}(\mathbf{E}_{ik}|\tilde{\mathbf{x}}_k) = \mathbf{W}_{i\cdot}^{(t)}\,\mathbb{E}(\mathbf{z}_k) + \boldsymbol{\mu}_i^{(t)}$

Find $\mathbb{E}(\mathbf{x}_k\mathbf{x}_k^\mathsf{T})$ given Observed Data:

Similarly, $\mathbb{E}(\mathbf{x}_k\mathbf{x}_k^\mathsf{T}) := \mathbb{E}(\mathbf{x}_k\mathbf{x}_k^\mathsf{T}|\tilde{\mathbf{x}}_k)$ and the derivation is carried out element-wisely. For any i-th and j-th dimension of $\mathbf{x}_k$, we focus on the matrix element $\mathbb{E}(\mathbf{x}_k\mathbf{x}_k^\mathsf{T}|\tilde{\mathbf{x}}_k;\Theta^{(t)})_{ij} = \mathbb{E}(\mathbf{X}_{ik}\mathbf{X}_{jk}|\tilde{\mathbf{x}}_k;\Theta^{(t)})$,

- if $\mathbf{O}_{ik} = \mathbf{O}_{jk} = 1$, $\mathbb{E}(\mathbf{X}_{ik}\mathbf{X}_{jk}|\tilde{\mathbf{x}}_k;\Theta^{(t)}) = \mathbf{X}_{ik}\mathbf{X}_{jk}$
- if $\mathbf{O}_{ik} = 0$ and $\mathbf{O}_{jk} = 1$, $\mathbb{E}(\mathbf{X}_{ik}\mathbf{X}_{jk}|\tilde{\mathbf{x}}_k;\Theta^{(t)}) = \mathbb{E}(\mathbf{X}_{ik}|\tilde{\mathbf{x}}_k;\Theta^{(t)})\mathbf{X}_{jk} = (\mathbf{W}_{i\cdot}^{(t)}\,\mathbb{E}(\mathbf{z}_k) + \boldsymbol{\mu}_i^{(t)})\mathbf{X}_{jk}$
- if $\mathbf{O}_{ik} = 1$ and $\mathbf{O}_{jk} = 0$, $\mathbb{E}(\mathbf{X}_{ik}\mathbf{X}_{jk}|\tilde{\mathbf{x}}_k;\Theta^{(t)}) = \mathbf{X}_{ik}\,\mathbb{E}(\mathbf{X}_{jk}|\tilde{\mathbf{x}}_k;\Theta^{(t)}) = \mathbf{X}_{ik}(\mathbf{W}_{j\cdot}^{(t)}\,\mathbb{E}(\mathbf{z}_k) + \boldsymbol{\mu}_j^{(t)})$
- if $\mathbf{O}_{ik} = \mathbf{O}_{jk} = 0$, $\mathbb{E}(\mathbf{X}_{ik}\mathbf{X}_{jk}|\tilde{\mathbf{x}}_k;\Theta^{(t)}) = \mathrm{Cov}(\mathbf{X}_{ik},\mathbf{X}_{jk}|\tilde{\mathbf{x}}_k;\Theta^{(t)}) + \mathbb{E}(\mathbf{X}_{ik}|\tilde{\mathbf{x}}_k;\Theta^{(t)})\,\mathbb{E}(\mathbf{X}_{jk}|\tilde{\mathbf{x}}_k;\Theta^{(t)})$
  $= \mathbf{W}_{i\cdot}^{(t)}\tilde{\mathbf{M}}_k^{(t)}\mathbf{W}_{j\cdot}^{(t)\mathsf{T}} + \boldsymbol{\Psi}_{ij}^{(t)} + (\mathbf{W}_{i\cdot}^{(t)}\,\mathbb{E}(\mathbf{z}_k) + \boldsymbol{\mu}_i^{(t)})(\mathbf{W}_{j\cdot}^{(t)}\,\mathbb{E}(\mathbf{z}_k) + \boldsymbol{\mu}_j^{(t)})$

Find $\mathbb{E}(\mathbf{z}_k\mathbf{x}_k^\mathsf{T})$ given Observed Data:

To perform the derivation element-wisely, recall $\mathbf{x}_k := \mathbf{X}_{\cdot k}$, and for the i-th dimension of $\mathbf{x}_k$, $\mathbb{E}(\mathbf{x}_k\mathbf{z}_k^\mathsf{T}|\tilde{\mathbf{x}}_k)_i = \mathbb{E}(\mathbf{X}_{\cdot k}\mathbf{z}_k^\mathsf{T}|\tilde{\mathbf{x}}_k)_i = \mathbb{E}(\mathbf{X}_{ik}\mathbf{z}_k^\mathsf{T}|\tilde{\mathbf{x}}_k)$,

- if $\mathbf{O}_{ik} = 1$, then $\mathbb{E}(\mathbf{X}_{ik}\mathbf{z}_k^\mathsf{T}|\tilde{\mathbf{x}}_k) = \mathbf{X}_{ik}(\mathbb{E}(\mathbf{z}_k|\tilde{\mathbf{x}}_k))^\mathsf{T} = \mathbf{X}_{ik}(\mathbb{E}(\mathbf{z}_k))^\mathsf{T}$
- if $\mathbf{O}_{ik} = 0$, then $\mathbb{E}(\mathbf{X}_{ik}\mathbf{z}_k^\mathsf{T}|\tilde{\mathbf{x}}_k) = \mathrm{Cov}(\mathbf{X}_{ik},\mathbf{z}_k^\mathsf{T}|\tilde{\mathbf{x}}_k) + \mathbb{E}(\mathbf{X}_{ik}|\tilde{\mathbf{x}}_k)\,\mathbb{E}(\mathbf{z}_k^\mathsf{T}|\tilde{\mathbf{x}}_k)$
  $= \mathrm{Cov}(\mathbf{W}_{i\cdot}\mathbf{z}_k + \boldsymbol{\mu}_i + \mathbf{E}_{ik}, \mathbf{z}_k^\mathsf{T}|\tilde{\mathbf{x}}_k) + \mathbb{E}(\mathbf{X}_{ik}|\tilde{\mathbf{x}}_k)\,\mathbb{E}(\mathbf{z}_k^\mathsf{T}|\tilde{\mathbf{x}}_k)$
  $= \mathbf{W}_{i\cdot}^{(t)}\tilde{\mathbf{M}}_k^{(t)} + (\mathbf{W}_{i\cdot}^{(t)}\,\mathbb{E}(\mathbf{z}_k) + \boldsymbol{\mu}_i^{(t)})(\mathbb{E}(\mathbf{z}_k))^\mathsf{T}$

1.1.5 EM Algorithm

Starting with certain initial values $\boldsymbol{\Theta}^{(0)} = (\mathbf{W}^{(0)}, \boldsymbol{\mu}^{(0)}, \boldsymbol{\Psi}^{(0)})$, we apply the following updates for E-step and then M-step iteratively until convergence. Assuming the t-th iteration is completed, at the $(t+1)$-th iteration, the two steps are performed as follows.

E-step:

For each sample $(k = 1, 2, \ldots, n)$, compute the following conditional expectations:

$$\mathbb{E}(\mathbf{z}_k) = \tilde{\mathbf{M}}_k^{(t)}\tilde{\mathbf{W}}_k^{(t)^\mathsf{T}}\tilde{\boldsymbol{\Psi}}_k^{(t)^{-1}}(\tilde{\mathbf{x}}_k - \tilde{\boldsymbol{\mu}}_k^{(t)})$$
$$\mathbb{E}(\mathbf{z}_k\mathbf{z}_k^\mathsf{T}) = \tilde{\mathbf{M}}_k^{(t)} + \mathbb{E}(\mathbf{z}_k)(\mathbb{E}(\mathbf{z}_k))^\mathsf{T}$$

Next, for each feature dimension $(i \in \{1, 2, \ldots, m\})$, compute:

$$\mathbb{E}(\mathbf{X}_{ik}) = \begin{cases} \mathbf{X}_{ik}, & \text{if } \mathbf{O}_{ik} = 1 \\ \mathbf{W}_{i\cdot}^{(t)}\,\mathbb{E}(\mathbf{z}_k) + \boldsymbol{\mu}_i^{(t)}, & \text{if } \mathbf{O}_{ik} = 0 \end{cases}$$
$$\mathbb{E}(\mathbf{X}_{ik}\mathbf{z}_k^\mathsf{T}) = \begin{cases} \mathbf{X}_{ik}(\mathbb{E}(\mathbf{z}_k))^\mathsf{T}, & \text{if } \mathbf{O}_{ik} = 1 \\ \mathbf{W}_{i\cdot}^{(t)}\tilde{\mathbf{M}}_k^{(t)} + (\mathbf{W}_{i\cdot}^{(t)}\,\mathbb{E}(\mathbf{z}_k) + \boldsymbol{\mu}_i^{(t)})(\mathbb{E}(\mathbf{z}_k))^\mathsf{T}, & \text{if } \mathbf{O}_{ik} = 0 \end{cases}$$

Then , for each pair of features ($i, j \in \{1, 2, \ldots, m\}$), compute:

$$\mathbb{E}(\mathbf{X}_{ik}\mathbf{X}_{jk}) = \begin{cases} \mathbf{X}_{ik}\mathbf{X}_{jk}, & \text{if } \mathbf{O}_{ik} = \mathbf{O}_{jk} = 1 \\ (\mathbf{W}_{i\cdot}^{(t)}\,\mathbb{E}(\mathbf{z}_k) + \boldsymbol{\mu}_i^{(t)})\mathbf{X}_{jk}, & \text{if } \mathbf{O}_{ik} = 0 \text{ and } \mathbf{O}_{jk} = 1 \\ \mathbf{X}_{ik}(\mathbf{W}_{j\cdot}^{(t)}\,\mathbb{E}(\mathbf{z}_k) + \boldsymbol{\mu}_j^{(t)}), & \text{if } \mathbf{O}_{ik} = 1 \text{ and } \mathbf{O}_{jk} = 0 \\ \mathbf{W}_{i\cdot}^{(t)}\tilde{\mathbf{M}}_k^{(t)}\mathbf{W}_{j\cdot}^{(t)\mathsf{T}} + \boldsymbol{\Psi}_{ij}^{(t)} & \\ +(\mathbf{W}_{i\cdot}^{(t)}\,\mathbb{E}(\mathbf{z}_k) + \boldsymbol{\mu}_i^{(t)})(\mathbf{W}_{j\cdot}^{(t)}\,\mathbb{E}(\mathbf{z}_k) + \boldsymbol{\mu}_j^{(t)}), & \text{if } \mathbf{O}_{ik} = \mathbf{O}_{jk} = 0 \end{cases}$$

M-step:
Update parameters $\boldsymbol{\mu}$ and $\mathbf{W}$ by:

$$\boldsymbol{\mu}^{(t+1)} = \frac{1}{n}\sum_{k=1}^{n}(\mathbb{E}(\mathbf{x}_k) - \mathbf{W}^{(t)}\,\mathbb{E}(\mathbf{z}_k))$$

$$\mathbf{W}^{(t+1)} = [\sum_{k=1}^{n}(\mathbb{E}(\mathbf{x}_k\mathbf{z}_k^{\mathsf{T}}) - \boldsymbol{\mu}^{(t)}(\mathbb{E}(\mathbf{z}_k))^{\mathsf{T}})][\sum_{k=1}^{n}\mathbb{E}(\mathbf{z}_k\mathbf{z}_k^{\mathsf{T}})]^{-1}$$

For parameter $\boldsymbol{\Psi}$:

$$\boldsymbol{\Psi}^{(t+1)} = \frac{1}{n}\sum_{k=1}^{n}\mathsf{Bdiag}_{\boldsymbol{\Psi}}[{\mathbf{G}_k^{(t)}}^{\mathsf{T}}],$$

where $\mathbf{G}_k^{(t)} = \mathbb{E}(\mathbf{x}_k\mathbf{x}_k^{\mathsf{T}}) + \boldsymbol{\mu}^{(t)}{\boldsymbol{\mu}^{(t)}}^{\mathsf{T}} + \mathbf{W}^{(t)}\,\mathbb{E}(\mathbf{z}_k\mathbf{z}_k^{\mathsf{T}}){\mathbf{W}^{(t)}}^{\mathsf{T}} - 2\,\mathbb{E}(\mathbf{x}_k){\boldsymbol{\mu}^{(t)}}^{\mathsf{T}} + 2\mathbf{W}^{(t)}\,\mathbb{E}(\mathbf{z}_k){\boldsymbol{\mu}^{(t)}}^{\mathsf{T}} - 2\mathbf{W}^{(t)}\,\mathbb{E}(\mathbf{z}_k\mathbf{x}_k^{\mathsf{T}})$.

1.1.6 Algorithm for Complete Data without Missing Values

When the data matrix $\mathbf{X}$ is complete without any missingness, the EM algorithm can be considerably simplified. Starting with certain initialization $\boldsymbol{\Theta}^{(0)} = (\mathbf{W}^{(0)}, \boldsymbol{\mu}^{(0)}, \boldsymbol{\Psi}^{(0)})$, suppose the t-th iteration is completed. At the $(t+1)$-th iteration, compute the following conditional expectations for the k-th subject:

$$\begin{aligned} \mathbb{E}(\mathbf{z}_k) &= \mathbf{M}^{(t)}{\mathbf{W}^{(t)}}^{\mathsf{T}}{\boldsymbol{\Psi}^{(t)}}^{-1}(\mathbf{x}_k - \boldsymbol{\mu}^{(t)}) \\ \mathbb{E}(\mathbf{z}_k\mathbf{z}_k^{\mathsf{T}}) &= \mathbf{M}^{(t)} + \mathbb{E}(\mathbf{z}_k)(\mathbb{E}(\mathbf{z}_k))^{\mathsf{T}} \\ \mathbb{E}(\mathbf{x}_k) &= \mathbf{x}_k \\ \mathbb{E}(\mathbf{x}_k\mathbf{x}_k^{\mathsf{T}}) &= \mathbf{x}_k\mathbf{x}_k^{\mathsf{T}} \\ \mathbb{E}(\mathbf{x}_k\mathbf{z}_k^{\mathsf{T}}) &= \mathbf{x}_k(\mathbb{E}(\mathbf{z}_k))^{\mathsf{T}} \end{aligned}$$

The M-step is identical to what we have presented in Section 1.1.5.

1.1.7 Parameter Initialization

(1) Mean Vector $\boldsymbol{\mu}$

If data matrix $\mathbf{X}$ is complete, $\boldsymbol{\mu}^{(0)}$ is initialized to be the sample mean due to its unbiasedness. However, if $\mathbf{X}$ is incomplete, then missing values are ignored while calculating the sample mean.

(2) Loading Matrix $\mathbf{W}$

We propose two options for initializing $\mathbf{W}^{(0)}$. First, we can initialize all elements of $\mathbf{W}^{(0)}$ with i.i.d. standard normal random variables. Alternatively, PCA or its variants [2] can be performed on the data matrix $\mathbf{X}$. Then, $\mathbf{W}^{(0)}$ is taken to be the learned matrix of variable loadings.

(3) Error Covariance Matrix $\boldsymbol{\Psi}$

If data matrix $\mathbf{X}$ is complete, $\boldsymbol{\Psi}^{(0)}$ is initialized to be a block diagonal matrix with each main-diagonal block being the sample covariance matrix $\mathbf{S}_{\mathbf{X}^{(r)}}$ of data $\mathbf{X}^{(r)}$ for the r-th modality:

$$\boldsymbol{\Psi}^{(0)} = \text{diag}(\mathbf{S}_{\mathbf{X}^{(1)}}, \mathbf{S}_{\mathbf{X}^{(2)}}, \ldots, \mathbf{S}_{\mathbf{X}^{(R)}}).$$

However, if $\mathbf{X}$ is incomplete, a random correlation matrix $\mathbf{R}_{\mathbf{X}^{(r)}}$ is generated for the r-th modality. In addition, we compute $\{s_i^{(r)}\}_{i\in I^{(r)}}$, the sample standard deviations of $\{\mathbf{X}_{i\cdot}^{(r)}\}_{i\in I^{(r)}}$, where $I^{(r)}$ is the index set of features in the r-th modality. Then,

$$\boldsymbol{\Psi}^{(0)} = \text{diag}(\mathbf{S}^*_{\mathbf{X}^{(1)}}, \mathbf{S}^*_{\mathbf{X}^{(2)}}, \ldots, \mathbf{S}^*_{\mathbf{X}^{(R)}}),$$

where $\mathbf{D}_{\mathbf{X}^{(r)}} = \text{diag}(\{s_i^{(r)}\}_{i\in I^{(r)}})$, and $\mathbf{S}^*_{\mathbf{X}^{(r)}} = \mathbf{D}_{\mathbf{X}^{(r)}}\mathbf{R}_{\mathbf{X}^{(r)}}\mathbf{D}_{\mathbf{X}^{(r)}}$.

1.1.8 Monitoring of Convergence

To assess the convergence of our EM algorithm for GPCCA, we focus on the latent variable matrix $\mathbf{Z}$. The algorithm stops when the following difference in $\mathbf{Z}$ between two consecutive steps (t and $t+1$) is smaller than a threshold τ

$$\text{RMSE}_{(t)}^{(t+1)} = \sqrt{\frac{1}{nd}\textstyle\sum_{i=1}^{d}\sum_{j=1}^{n}(\mathbf{Z}_{i,j}^{(t+1)} - \mathbf{Z}_{i,j}^{(t)})^2} < \tau.$$

1.1.9 Improvement of Computational Efficiency

In this section, we introduce several implementation adjustments to improve the computational efficiency of our algorithm.

Adaptive Inversion of Partial Error Covariance

For large-sclae data, finding the inverse of $\tilde{\boldsymbol{\Psi}}_k$ can be computationally demanding.

Given subject k, suppose $h_k(\cdot)$ is a function that rearrange the elements of a square matrix based on the missing patterns:

$$h_k(\boldsymbol{\Psi}) = \begin{pmatrix} \boldsymbol{\Psi}_{\text{m,m}} & \boldsymbol{\Psi}_{\text{m,o}} \\ \boldsymbol{\Psi}_{\text{o,m}} & \boldsymbol{\Psi}_{\text{o,o}} \end{pmatrix}, \text{ where } \begin{cases} \boldsymbol{\Psi}_{\text{o,o}} := \{\boldsymbol{\Psi}_{ij}\}_{i,j\in\{g:\mathbf{O}_{gk}=1\}} \\ \boldsymbol{\Psi}_{\text{o,m}} := \{\boldsymbol{\Psi}_{ij}\}_{i\in\{g:\mathbf{O}_{gk}=1\},j\in\{g:\mathbf{O}_{gk}=0\}} \\ \boldsymbol{\Psi}_{\text{m,o}} := \{\boldsymbol{\Psi}_{ij}\}_{i\in\{g:\mathbf{O}_{gk}=0\},j\in\{g:\mathbf{O}_{gk}=1\}} \\ \boldsymbol{\Psi}_{\text{m,m}} := \{\boldsymbol{\Psi}_{ij}\}_{i,j\in\{g:\mathbf{O}_{gk}=0\}} \end{cases}.$$

Similarly, $h_k(\cdot)$ can be applied to $\mathbf{\Psi}^{-1}$. Each block of $h_k(\mathbf{\Psi}^{-1})$ is equivalent to the corresponding submatrix of $\mathbf{\Psi}^{-1}$:

$$h_k(\mathbf{\Psi}^{-1}) = \begin{pmatrix} (\mathbf{\Psi}^{-1})_{\text{m,m}} & (\mathbf{\Psi}^{-1})_{\text{m,o}} \\ (\mathbf{\Psi}^{-1})_{\text{o,m}} & (\mathbf{\Psi}^{-1})_{\text{o,o}} \end{pmatrix}, \text{ where } \begin{cases} (\mathbf{\Psi}^{-1})_{\text{o,o}} := \{\mathbf{\Psi}^{-1}_{ij}\}_{i,j\in\{g:\mathbf{O}_{gk}=1\}} \\ (\mathbf{\Psi}^{-1})_{\text{o,m}} := \{\mathbf{\Psi}^{-1}_{ij}\}_{i\in\{g:\mathbf{O}_{gk}=1\},j\in\{g:\mathbf{O}_{gk}=0\}} \\ (\mathbf{\Psi}^{-1})_{\text{m,o}} := \{\mathbf{\Psi}^{-1}_{ij}\}_{i\in\{g:\mathbf{O}_{gk}=0\},j\in\{g:\mathbf{O}_{gk}=1\}} \\ (\mathbf{\Psi}^{-1})_{\text{m,m}} := \{\mathbf{\Psi}^{-1}_{ij}\}_{i,j\in\{g:\mathbf{O}_{gk}=0\}} \end{cases}.$$

Once $\mathbf{\Psi}$ is ready from either initialization or the most recent update, we compute its inverse $\mathbf{\Psi}^{-1}$. Then, for each subject k, $\tilde{\mathbf{\Psi}}_k^{-1}$ can be derived adaptively as follows (using Corollary 1 below):

$$\tilde{\mathbf{\Psi}}_k^{-1} = (\mathbf{\Psi}_{\text{o,o}})^{-1} = (\mathbf{\Psi}^{-1})_{\text{o,o}} - (\mathbf{\Psi}^{-1})_{\text{o,m}}[(\mathbf{\Psi}^{-1})_{\text{m,m}}]^{-1}(\mathbf{\Psi}^{-1})_{\text{m,o}}.$$

For high-dimensional data, using the above derivation is generally much faster than computing $\tilde{\mathbf{\Psi}}_k^{-1}$ directly.

Corollary 1 If a given invertible matrix is symmetric with invertible block $\mathbf{A}$, then the following relationship holds, where $\mathbf{S} = \mathbf{D} - \mathbf{C}\mathbf{A}^{-1}\mathbf{C}^\mathsf{T}$. [3]

$$\begin{pmatrix} \mathbf{A} & \mathbf{C}^\mathsf{T} \\ \mathbf{C} & \mathbf{D} \end{pmatrix}^{-1} = \begin{pmatrix} \mathbf{A}^{-1} + \mathbf{A}^{-1}\mathbf{C}^\mathsf{T}\mathbf{S}^{-1}\mathbf{C}\mathbf{A}^{-1} & -\mathbf{A}^{-1}\mathbf{C}^\mathsf{T}\mathbf{S}^{-1} \\ -\mathbf{S}^{-1}\mathbf{C}\mathbf{A}^{-1} & \mathbf{S}^{-1} \end{pmatrix}$$

Proof:

For a given square matrix $\mathbf{A}$, $h_k(\mathbf{A})$ is realized through $h_k(\mathbf{A}) = \mathbf{P}\mathbf{A}\mathbf{P}^\mathsf{T}$ where $\mathbf{P}$ is a permutation matrix that rearrange the rows of missing dimensions ahead of the rows of observed dimensions. Suppose $\mathbf{A}$ is also invertible, and then $h_k(\mathbf{A}^{-1}) = \mathbf{P}\mathbf{A}^{-1}\mathbf{P}^\mathsf{T}$. Since every permutation matrix is orthogonal, $\mathbf{P}^{-1} = \mathbf{P}^\mathsf{T}$. Hence, $(h_k(\mathbf{A}))^{-1} = (\mathbf{P}\mathbf{A}\mathbf{P}^\mathsf{T})^{-1} = (\mathbf{P}^\mathsf{T})^{-1}\mathbf{A}^{-1}\mathbf{P}^{-1} = \mathbf{P}\mathbf{A}^{-1}\mathbf{P}^\mathsf{T} = h_k(\mathbf{A}^{-1})$. Due to $(h_k(\mathbf{A}))^{-1} = h_k(\mathbf{A}^{-1})$ and **Corollary 1**, we have the following two relationships.

$$\begin{pmatrix} (\mathbf{\Psi}^{-1})_{\text{m,m}} & (\mathbf{\Psi}^{-1})_{\text{m,o}} \\ (\mathbf{\Psi}^{-1})_{\text{o,m}} & (\mathbf{\Psi}^{-1})_{\text{o,o}} \end{pmatrix} = h_k(\mathbf{\Psi}^{-1}) = \begin{pmatrix} \mathbf{A} & \mathbf{C}^\mathsf{T} \\ \mathbf{C} & \mathbf{D} \end{pmatrix}$$

$$\begin{pmatrix} \mathbf{\Psi}_{\text{m,m}} & \mathbf{\Psi}_{\text{m,o}} \\ \mathbf{\Psi}_{\text{o,m}} & \mathbf{\Psi}_{\text{o,o}} \end{pmatrix} = h_k(\mathbf{\Psi}) = (h_k(\mathbf{\Psi}^{-1}))^{-1} = \begin{pmatrix} \mathbf{A} & \mathbf{C}^\mathsf{T} \\ \mathbf{C} & \mathbf{D} \end{pmatrix}^{-1} = \begin{pmatrix} \mathbf{A}^{-1} + \mathbf{V}^\mathsf{T}\mathbf{S}\mathbf{V} & -\mathbf{V}^\mathsf{T} \\ -\mathbf{V} & \mathbf{S}^{-1} \end{pmatrix}$$

Concisely, rewrite $\mathbf{V} = \mathbf{S}^{-1}\mathbf{C}\mathbf{A}^{-1}$. But we focus on comparing the bottom-right blocks $\mathbf{\Psi}_{\text{o,o}} = \mathbf{S}^{-1}$. If follows that $\tilde{\mathbf{\Psi}}_k^{-1} = (\mathbf{\Psi}_{\text{o,o}})^{-1} = \mathbf{S} = \mathbf{D} - \mathbf{C}\mathbf{A}^{-1}\mathbf{C}^\mathsf{T} = (\mathbf{\Psi}^{-1})_{\text{o,o}} - (\mathbf{\Psi}^{-1})_{\text{o,m}}[(\mathbf{\Psi}^{-1})_{\text{m,m}}]^{-1}(\mathbf{\Psi}^{-1})_{\text{m,o}}$.

Blockwise Matrix Inversion by Modality

One of the model assumptions of GPCCA is the block diagonal structure of $\mathbf{\Psi}$:

$$\mathbf{\Psi} = \text{diag}(\mathbf{\Psi}^{(1)}, \mathbf{\Psi}^{(2)}, \ldots, \mathbf{\Psi}^{(R)}) \Rightarrow \mathbf{\Psi}^{-1} = \text{diag}({\mathbf{\Psi}^{(1)}}^{-1}, {\mathbf{\Psi}^{(2)}}^{-1}, \ldots, {\mathbf{\Psi}^{(R)}}^{-1}).$$

In addition to $\mathbf{\Psi}$ itself, finding the inverse of the partial covariance matrix $\tilde{\mathbf{\Psi}}_k$ can also be performed by modality:

$${\tilde{\mathbf{\Psi}}_k^{(r)}}^{-1} = (\mathbf{\Psi}^{(r)}_{\text{o,o}})^{-1} = ({\mathbf{\Psi}^{(r)}}^{-1})_{\text{o,o}} - ({\mathbf{\Psi}^{(r)}}^{-1})_{\text{o,m}}[({\mathbf{\Psi}^{(r)}}^{-1})_{\text{m,m}}]^{-1}({\mathbf{\Psi}^{(r)}}^{-1})_{\text{m,o}}.$$

1.2 Regularization in GPCCA Model

As the dimensionality of features increases, the estimation of the error covariance matrix $\mathbf{\Psi}$ becomes increasingly unstable. Moreover, in many real-world applications, it is reasonable to assume that each feature is directly associated with only a limited subset of other features within each modality. To address these challenges, we introduce ridge regularization on the error covariance matrix to enhance the stability of its estimation.

Suppose we use $\mathbf{R}$ to denote the error correlation matrix, then the error covariance matrix $\mathbf{\Psi}$ can be factorized as $\mathbf{\Psi} = f(\mathbf{\Psi}_d, \mathbf{R}) = \mathbf{\Psi}_d^{\frac{1}{2}}\mathbf{R}\mathbf{\Psi}_d^{\frac{1}{2}}$, where $\mathbf{\Psi}_d$ is a diagonal matrix with positive values.

As previously derived, the expected complete log-likelihood can be represented in the form below:

$$\mathbb{E}[\ell_{\mathsf{c}}(\mathbf{\Theta})] \propto \mathcal{Q}(\mathbf{\Theta}) = -\frac{n}{2}\ln|\mathbf{\Psi}| - \frac{1}{2}\sum_{k=1}^{n}\mathsf{tr}(\mathbf{\Psi}^{-1}\mathbf{G}_k) - \frac{1}{2}\sum_{k=1}^{n}\mathsf{tr}(\mathbb{E}(\mathbf{z}_k\mathbf{z}_k^{\mathsf{T}})).$$

The ridge estimator of the error correlation matrix $\mathbf{R}$ is the maximum penalized likelihood estimator (MPLE) with a penalty term proportional to $-\mathsf{tr}(\mathbf{R}^{-1})$ [4]. Therefore, to obtain the ridge estimator, we modify $\mathcal{Q}(\mathbf{\Theta})$ by adding a penalty term as follows:

$$\begin{aligned}\mathcal{Q}_c(\boldsymbol{R}) &= \mathcal{Q}(\mathbf{\Theta}) - \frac{c}{2}\mathsf{tr}(\mathbf{R}^{-1})\\ &\propto -\frac{n}{2}\ln|\mathbf{\Psi}| - \frac{1}{2}\sum_{k=1}^{n}\mathsf{tr}(\mathbf{\Psi}^{-1}\mathbf{G}_k) - \frac{c}{2}\mathsf{tr}(\mathbf{R}^{-1}),\end{aligned}$$

where $c > 0$ and terms not dependent on $\boldsymbol{R}$ are ignored. Next, to maximize $\mathcal{Q}_c(\boldsymbol{R})$, we investigate the minimum of $-2\mathcal{Q}_c(\boldsymbol{R})$. We have

$$\begin{aligned}-2\mathcal{Q}_c(\boldsymbol{R}) &\propto n\ln|\mathbf{\Psi}| + \sum_{k=1}^{n}\mathsf{tr}(\mathbf{\Psi}^{-1}\mathbf{G}_k) + c\mathsf{tr}(\mathbf{R}^{-1})\\ &= n\ln|\mathbf{\Psi}_d^{\frac{1}{2}}\mathbf{R}\mathbf{\Psi}_d^{\frac{1}{2}}| + \sum_{k=1}^{n}\mathsf{tr}(\mathbf{\Psi}^{-1}\mathbf{G}_k) + n\mathsf{tr}(\frac{c}{n}\mathbf{R}^{-1})\\ &= n\ln|\mathbf{\Psi}_d^{\frac{1}{2}}||\mathbf{R}||\mathbf{\Psi}_d^{\frac{1}{2}}| + \mathsf{tr}(\sum_{k=1}^{n}\mathbf{\Psi}^{-1}\mathbf{G}_k) + n\mathsf{tr}(\frac{c}{n}\mathbf{R}^{-1})\\ &= n\ln|\mathbf{\Psi}_d^{\frac{1}{2}}||\mathbf{\Psi}_d^{\frac{1}{2}}| + n\ln|\mathbf{R}| + n\mathsf{tr}(\mathbf{\Psi}^{-1}\frac{1}{n}\sum_{k=1}^{n}\mathbf{G}_k) + n\mathsf{tr}(\frac{c}{n}\mathbf{R}^{-1})\\ &= n\ln|\mathbf{\Psi}_d| + n\ln|\mathbf{R}| + n\mathsf{tr}(\mathbf{\Psi}^{-1}\frac{1}{n}\sum_{k=1}^{n}\mathbf{G}_k) + n\mathsf{tr}(\frac{c}{n}\mathbf{R}^{-1})\\ &= n\ln|\mathbf{\Psi}_d| + n\ln|\mathbf{R}| + n\mathsf{tr}(\mathbf{\Psi}^{-1}\mathbf{S}) + n\mathsf{tr}(\frac{c}{n}\mathbf{R}^{-1})\\ &= n\ln|\mathbf{\Psi}_d| + n\ln|\mathbf{R}| + n\mathsf{tr}(\mathbf{\Psi}_d^{-\frac{1}{2}}\mathbf{R}^{-1}\mathbf{\Psi}_d^{-\frac{1}{2}}\mathbf{S}) + n\mathsf{tr}(\frac{c}{n}\mathbf{R}^{-1})\\ &= n\ln|\mathbf{\Psi}_d| + n\ln|\mathbf{R}| + n\mathsf{tr}(\mathbf{\Psi}_d^{-\frac{1}{2}}\mathbf{S}\mathbf{\Psi}_d^{-\frac{1}{2}}\mathbf{R}^{-1}) + n\mathsf{tr}(\frac{c}{n}\mathbf{R}^{-1})\\ &= n\ln|\mathbf{\Psi}_d| + n\ln|\mathbf{R}| + n\mathsf{tr}(\mathbf{\Psi}_d^{-\frac{1}{2}}\mathbf{S}\mathbf{\Psi}_d^{-\frac{1}{2}}\mathbf{R}^{-1} + \frac{c}{n}\mathbf{R}^{-1})\end{aligned}$$

$$= n\ln|\mathbf{\Psi}_d| + n\ln|\mathbf{R}| + n\mathsf{tr}[(\mathbf{\Psi}_d^{-\frac{1}{2}}\mathbf{S}\mathbf{\Psi}_d^{-\frac{1}{2}} + \frac{c}{n}\mathbf{I})\mathbf{R}^{-1}]$$
$$= n\ln|\mathbf{\Psi}_d| + n\ln|\mathbf{R}| + n\mathsf{tr}(\mathbf{K}\mathbf{R}^{-1}).$$

Hence, $-2\mathcal{Q}_c(\boldsymbol{R}) \propto \ln|\mathbf{\Psi}_d| + \ln|\mathbf{R}| + \mathsf{tr}(\mathbf{K}\mathbf{R}^{-1})$, where $\mathbf{S} := \frac{1}{n}\sum_{k=1}^{n}\mathbf{G}_k$, and $\mathbf{K} := \mathbf{\Psi}_d^{-\frac{1}{2}}\mathbf{S}\mathbf{\Psi}_d^{-\frac{1}{2}} + \frac{c}{n}\mathbf{I}$. With $\mathbf{R} = \mathsf{diag}(\mathbf{R}^{(1)}, \mathbf{R}^{(2)}, \ldots, \mathbf{R}^{(R)})$ and $\mathbf{R}^{-1} = \mathsf{diag}(\mathbf{R}^{(1)^{-1}}, \mathbf{R}^{(2)^{-1}}, \ldots, \mathbf{R}^{(R)^{-1}})$, we rewrite $\mathcal{Q}_c(\boldsymbol{R}) := \mathcal{Q}_c(\mathbf{R}^{(1)}, \mathbf{R}^{(2)}, \ldots, \mathbf{R}^{(R)})$ and can show that

$$\begin{aligned}
-2\mathcal{Q}_c(\boldsymbol{R}) &= -2\mathcal{Q}_c(\mathbf{R}^{(1)}, \mathbf{R}^{(2)}, \ldots, \mathbf{R}^{(R)}) \\
&\propto \ln|\mathbf{\Psi}_d| + \ln|\mathbf{R}| + \mathsf{tr}(\mathbf{K}\mathbf{R}^{-1}) \\
&= \ln|\mathbf{\Psi}_d| + \ln\prod_{r=1}^{R}|\mathbf{R}^{(r)}| + \mathsf{tr}((\mathbf{\Psi}_d^{-\frac{1}{2}}\frac{1}{n}\sum_{k=1}^{n}\mathbf{G}_k\mathbf{\Psi}_d^{-\frac{1}{2}} + \frac{c}{n}\mathbf{I})\mathbf{R}^{-1}) \\
&= \ln|\mathbf{\Psi}_d| + \sum_{r=1}^{R}\ln|\mathbf{R}^{(r)}| + \sum_{r=1}^{R}\mathsf{tr}((\mathbf{\Psi}_d^{(r)^{-\frac{1}{2}}}\frac{1}{n}\sum_{k=1}^{n}\mathbf{G}_k^{(r)}\mathbf{\Psi}_d^{(r)^{-\frac{1}{2}}} + \frac{c}{n}\mathbf{I})\mathbf{R}^{(r)^{-1}}) \\
&= \ln|\mathbf{\Psi}_d| + \sum_{r=1}^{R}(\ln|\mathbf{R}^{(r)}| + \mathsf{tr}(\mathbf{K}^{(r)}\mathbf{R}^{(r)^{-1}})),
\end{aligned}$$

where $\mathbf{K}^{(r)} := \frac{1}{n}\mathbf{\Psi}_d^{(r)^{-\frac{1}{2}}}\sum_{k=1}^{n}\mathbf{G}_k^{(r)}\mathbf{\Psi}_d^{(r)^{-\frac{1}{2}}} + \frac{c}{n}\mathbf{I}$. Since

$$\frac{\partial(-2\mathcal{Q}_c(\mathbf{R}^{(1)}, \mathbf{R}^{(2)}, \ldots, \mathbf{R}^{(R)}))}{\partial\mathbf{R}^{(r)}} \propto \mathbf{R}^{(r)^{-1}}(\mathbf{I} - \mathbf{K}^{(r)}\mathbf{R}^{(r)^{-1}}),$$

$-2\mathcal{Q}_c(\mathbf{R}^{(1)}, \mathbf{R}^{(2)}, \ldots, \mathbf{R}^{(R)})$ is minimized when when $\mathbf{K}^{(r)}\mathbf{R}^{(r)^{-1}} = \mathbf{I}$, i.e., $\mathbf{R}^{(r)} = \mathbf{K}^{(r)}$ $(r = 1, \ldots, R)$. Therefore, the ridge estimator of the correlation matrix $\mathbf{R}$ can be initially written as follows.

$$\hat{\mathbf{R}}_{\mathsf{ridge}} = \mathsf{diag}(\mathbf{K}^{(1)}(\mathbf{\Psi}_d), \mathbf{K}^{(2)}(\mathbf{\Psi}_d), \ldots, \mathbf{K}^{(R)}(\mathbf{\Psi}_d)) = \frac{1}{n}\mathbf{\Psi}_d^{-\frac{1}{2}}\sum_{k=1}^{n}\mathsf{Bdiag}_{\mathbf{\Psi}}[\mathbf{G}_k]\mathbf{\Psi}_d^{-\frac{1}{2}} + \frac{c}{n}\mathbf{I}.$$

Since all diagonal elements of $\hat{\mathbf{R}}_{\mathsf{ridge}}$ are 1, we require

$$\begin{aligned}
\mathsf{Diag}[\hat{\mathbf{R}}_{\mathsf{ridge}}] &= \mathbf{\Psi}_d^{-\frac{1}{2}}\frac{1}{n}\sum_{k=1}^{n}\mathsf{Diag}[\mathsf{Bdiag}_{\mathbf{\Psi}}[\mathbf{G}_k]]\mathbf{\Psi}_d^{-\frac{1}{2}} + \frac{c}{n}\mathbf{I} \\
&= \mathbf{\Psi}_d^{-\frac{1}{2}}\frac{1}{n}\sum_{k=1}^{n}\mathsf{Diag}[\mathbf{G}_k]\mathbf{\Psi}_d^{-\frac{1}{2}} + \frac{c}{n}\mathbf{I} \\
&= \mathbf{\Psi}_d^{-\frac{1}{2}}\mathsf{Diag}[\frac{1}{n}\sum_{k=1}^{n}\mathbf{G}_k]\mathbf{\Psi}_d^{-\frac{1}{2}} + \frac{c}{n}\mathbf{I} \\
&= \mathbf{\Psi}_d^{-\frac{1}{2}}\mathsf{Diag}[\mathbf{S}]\mathbf{\Psi}_d^{-\frac{1}{2}} + \frac{c}{n}\mathbf{I} = \mathbf{I}.
\end{aligned}$$

This leads to $\mathsf{Diag}[\mathbf{S}] = (1 - \frac{c}{n})\mathbf{\Psi}_d$, where $c \in (0, n)$ due to non-negative variances. Let $\lambda = 1 - \frac{c}{n}$, then $\mathbf{\Psi}_d = \lambda^{-1}\mathsf{Diag}[\mathbf{S}]$. The estimated variances are updated as $\hat{\mathbf{\Psi}}_{d,\mathsf{ridge}} = \lambda^{-1}\mathsf{Diag}[\mathbf{S}]$.

Then,

$$
\begin{aligned}
\hat{\mathbf{R}}_{\mathsf{ridge}} &= \hat{\mathbf{\Psi}}_{d,\mathsf{ridge}}^{-\frac{1}{2}} \frac{1}{n}\sum_{k=1}^{n} \mathsf{Bdiag}_{\mathbf{\Psi}}[\mathbf{G}_k]\hat{\mathbf{\Psi}}_{d,\mathsf{ridge}}^{-\frac{1}{2}} + (1-\lambda)\mathbf{I} \\
&= (\lambda^{-1}\mathsf{Diag}[\mathbf{S}])^{-\frac{1}{2}} \frac{1}{n}\sum_{k=1}^{n} \mathsf{Bdiag}_{\mathbf{\Psi}}[\mathbf{G}_k](\lambda^{-1}\mathsf{Diag}[\mathbf{S}])^{-\frac{1}{2}} + (1-\lambda)\mathbf{I} \\
&= \lambda\mathsf{Diag}[\mathbf{S}]^{-\frac{1}{2}} \frac{1}{n}\sum_{k=1}^{n} \mathsf{Bdiag}_{\mathbf{\Psi}}[\mathbf{G}_k]\mathsf{Diag}[\mathbf{S}]^{-\frac{1}{2}} + (1-\lambda)\mathbf{I},
\end{aligned}
$$

where $\lambda \in (0,1)$.

Suppose we use $\hat{\mathbf{R}}$ to denote the original estimator of $\mathbf{R}$ without introducing the ridge regularization, below we show that $\hat{\mathbf{R}}_{\mathsf{ridge}} = \lambda\hat{\mathbf{R}} + (1-\lambda)\mathbf{I}$. For the estimation without ridge regularization, we find the MLE of $(\mathbf{\Psi}_d, \mathbf{R})$ jointly by setting

$$
\begin{cases}
\dfrac{\partial \mathcal{Q}(\mathbf{\Theta})}{\partial \mathbf{\Psi}_d} = \mathbf{0} \\
\dfrac{\partial \mathcal{Q}(\mathbf{\Theta})}{\partial \mathbf{R}} = \mathbf{0}
\end{cases}.
$$

With $\mathbf{\Psi} = \mathbf{\Psi}_d^{\frac{1}{2}}\mathbf{R}\mathbf{\Psi}_d^{\frac{1}{2}}$ and $\mathbf{\Psi}^{-1} = \mathbf{\Psi}_d^{-\frac{1}{2}}\mathbf{R}^{-1}\mathbf{\Psi}_d^{-\frac{1}{2}}$, $\mathcal{Q}(\mathbf{\Theta})$ can be represented as follows.

$$
\begin{aligned}
\mathcal{Q}(\mathbf{\Theta}) &= -\frac{n}{2}\ln|\mathbf{\Psi}| - \frac{1}{2}\sum_{k=1}^{n}\mathsf{tr}(\mathbf{\Psi}^{-1}\mathbf{G}_k) - \frac{1}{2}\sum_{k=1}^{n}\mathsf{tr}(\mathbb{E}(\mathbf{z}_k\mathbf{z}_k^{\mathsf{T}})) \\
&= -\frac{n}{2}\ln|\mathbf{\Psi}_d^{\frac{1}{2}}\mathbf{R}\mathbf{\Psi}_d^{\frac{1}{2}}| - \frac{1}{2}\sum_{k=1}^{n}\mathsf{tr}((\mathbf{\Psi}_d^{\frac{1}{2}}\mathbf{R}\mathbf{\Psi}_d^{\frac{1}{2}})^{-1}\mathbf{G}_k) - \frac{1}{2}\sum_{k=1}^{n}\mathsf{tr}(\mathbb{E}(\mathbf{z}_k\mathbf{z}_k^{\mathsf{T}})) \\
&= -\frac{n}{2}\ln|\mathbf{R}| - \frac{n}{2}\ln|\mathbf{\Psi}_d| - \frac{1}{2}\sum_{k=1}^{n}\mathsf{tr}(\mathbf{\Psi}_d^{-\frac{1}{2}}\mathbf{R}^{-1}\mathbf{\Psi}_d^{-\frac{1}{2}}\mathbf{G}_k) - \frac{1}{2}\sum_{k=1}^{n}\mathsf{tr}(\mathbb{E}(\mathbf{z}_k\mathbf{z}_k^{\mathsf{T}}))
\end{aligned}
$$

Taking partial derivative of $\mathcal{Q}(\mathbf{\Theta})$ w.r.t. marginal variance $\Psi_{d,i}$ of the i-th feature gives the following

$$
\begin{aligned}
\frac{\partial \mathcal{Q}(\mathbf{\Theta})}{\partial \Psi_{d,i}} &= -\frac{n}{2}\frac{\partial}{\partial \Psi_{d,i}}\ln|\mathbf{\Psi}_d| - \frac{1}{2}\sum_{k=1}^{n}\frac{\partial}{\partial \Psi_{d,i}}\mathsf{tr}(\mathbf{\Psi}_d^{-\frac{1}{2}}\mathbf{R}^{-1}\mathbf{\Psi}_d^{-\frac{1}{2}}\mathbf{G}_k) \\
&= -\frac{n}{2}\frac{\partial}{\partial \Psi_{d,i}}\ln\prod_{i=1}^{m}\Psi_{d,i} - \frac{1}{2}\sum_{k=1}^{n}\frac{\partial}{\partial \Psi_{d,i}}\sum_{i=1}^{m}(\Psi_{d,i}^{-\frac{1}{2}}\cdot 1\cdot \Psi_{d,i}^{-\frac{1}{2}}\cdot \mathbf{G}_{k,ii}) \\
&= -\frac{n}{2}\frac{\partial}{\partial \Psi_{d,i}}\sum_{i=1}^{m}\ln\Psi_{d,i} - \frac{1}{2}\sum_{k=1}^{n}\frac{\partial}{\partial \Psi_{d,i}}\sum_{i=1}^{m}(\Psi_{d,i}^{-1}\mathbf{G}_{k,ii}) \\
&= -\frac{n}{2}\Psi_{d,i}^{-1} - \frac{1}{2}\sum_{k=1}^{n}(-\Psi_{d,i}^{-2}\mathbf{G}_{k,ii})
\end{aligned}
$$

Setting $\dfrac{\partial \mathcal{Q}(\mathbf{\Theta})}{\partial \Psi_{d,i}} = 0$ gives the stationary point of $\mathcal{Q}(\mathbf{\Theta})$ w.r.t. $\Psi_{d,i}$:

$$
\hat{\Psi}_{d,i} = \frac{1}{n}\sum_{k=1}^{n}\mathbf{G}_{k,ii}.
$$

Then, combining the stationary point w.r.t. $\Psi_{d,i}$ across all features ($i = 1, \ldots, m$) gives the stationary point w.r.t. $\mathbf{\Psi}_d$:

$$\begin{aligned}
\hat{\mathbf{\Psi}}_d &= \text{diag}(\Psi_{d,1}, \Psi_{d,2}, \ldots, \Psi_{d,m}) \\
&= \text{diag}(\frac{1}{n}\sum_{k=1}^{n} \mathbf{G}_{k,11}, \frac{1}{n}\sum_{k=1}^{n} \mathbf{G}_{k,22}, \ldots, \frac{1}{n}\sum_{k=1}^{n} \mathbf{G}_{k,mm}) \\
&= \frac{1}{n}\sum_{k=1}^{n} \text{diag}(\mathbf{G}_{k,11}, \mathbf{G}_{k,22}, \ldots, \mathbf{G}_{k,mm}) \\
&= \frac{1}{n}\sum_{k=1}^{n} \text{Diag}[\mathbf{G}_k] = \text{Diag}[\frac{1}{n}\sum_{k=1}^{n} \mathbf{G}_k] = \text{Diag}[\mathbf{S}].
\end{aligned}$$

Then, in order to find the stationary point for the error correlation matrix $\mathbf{R}$, $\mathcal{Q}(\mathbf{\Theta})$ can be alternatively represented as follows.

$$\begin{aligned}
\mathcal{Q}(\mathbf{\Theta}) &= -\frac{n}{2} \ln \prod_{r=1}^{R} |\mathbf{\Psi}^{(r)}| - \frac{1}{2}\sum_{k=1}^{n}\sum_{r=1}^{R} \text{tr}(\mathbf{\Psi}^{(r)^{-1}} \mathbf{G}_k^{(r)}) - \frac{1}{2}\sum_{k=1}^{n} \text{tr}(\mathbb{E}(\mathbf{z}_k \mathbf{z}_k^{\mathsf{T}})) \\
&= -\frac{n}{2}\sum_{r=1}^{R} \ln |\mathbf{\Psi}^{(r)}| - \frac{1}{2}\sum_{r=1}^{R}\sum_{k=1}^{n} \text{tr}(\mathbf{\Psi}^{(r)^{-1}} \mathbf{G}_k^{(r)}) - \frac{1}{2}\sum_{k=1}^{n} \text{tr}(\mathbb{E}(\mathbf{z}_k \mathbf{z}_k^{\mathsf{T}})) \\
&= -\frac{n}{2}\sum_{r=1}^{R} \ln |\mathbf{\Psi}_d^{(r)^{\frac{1}{2}}} \mathbf{R}^{(r)} \mathbf{\Psi}_d^{(r)^{\frac{1}{2}}}| - \frac{1}{2}\sum_{r=1}^{R}\sum_{k=1}^{n} \text{tr}(\mathbf{\Psi}_d^{(r)^{-\frac{1}{2}}} \mathbf{R}^{(r)^{-1}} \mathbf{\Psi}_d^{(r)^{-\frac{1}{2}}} \mathbf{G}_k^{(r)}) - \frac{1}{2}\sum_{k=1}^{n} \text{tr}(\mathbb{E}(\mathbf{z}_k \mathbf{z}_k^{\mathsf{T}})) \\
&= -n\sum_{r=1}^{R} \ln |\mathbf{\Psi}_d^{(r)^{\frac{1}{2}}}| - \frac{n}{2}\sum_{r=1}^{R} \ln |\mathbf{R}^{(r)}| - \frac{1}{2}\sum_{r=1}^{R}\sum_{k=1}^{n} \text{tr}(\mathbf{\Psi}_d^{(r)^{-\frac{1}{2}}} \mathbf{R}^{(r)^{-1}} \mathbf{\Psi}_d^{(r)^{-\frac{1}{2}}} \mathbf{G}_k^{(r)}) - \frac{1}{2}\sum_{k=1}^{n} \text{tr}(\mathbb{E}(\mathbf{z}_k \mathbf{z}_k^{\mathsf{T}}))
\end{aligned}$$

Taking partial derivative of $\mathcal{Q}(\mathbf{\Theta})$ w.r.t. error correlation matrix $\mathbf{R}^{(r)}$ of the r-th modality gives the following

$$\begin{aligned}
\frac{\partial \mathcal{Q}(\mathbf{\Theta})}{\partial \mathbf{R}^{(r)}} &= -\frac{n}{2}\frac{\partial}{\partial \mathbf{R}^{(r)}} \ln |\mathbf{R}^{(r)}| - \frac{1}{2}\sum_{k=1}^{n} \frac{\partial}{\partial \mathbf{R}^{(r)}} \text{tr}(\mathbf{\Psi}_d^{(r)^{-\frac{1}{2}}} \mathbf{R}^{(r)^{-1}} \mathbf{\Psi}_d^{(r)^{-\frac{1}{2}}} \mathbf{G}_k^{(r)}) \\
&= -\frac{n}{2}\mathbf{R}^{(r)^{-1}} - \frac{1}{2}\sum_{k=1}^{n} (-\mathbf{R}^{(r)^{-1}} \mathbf{\Psi}_d^{(r)^{-\frac{1}{2}}} \mathbf{G}_k^{(r)} \mathbf{\Psi}_d^{(r)^{-\frac{1}{2}}} \mathbf{R}^{(r)^{-1}})^{\mathsf{T}} \\
&= -\frac{n}{2}\mathbf{R}^{(r)^{-1}} + \frac{1}{2}\mathbf{R}^{(r)^{-1}} \mathbf{\Psi}_d^{(r)^{-\frac{1}{2}}} (\sum_{k=1}^{n} \mathbf{G}_k^{(r)^{\mathsf{T}}}) \mathbf{\Psi}_d^{(r)^{-\frac{1}{2}}} \mathbf{R}^{(r)^{-1}}.
\end{aligned}$$

Setting $\frac{\partial \mathcal{Q}(\mathbf{\Theta})}{\partial \mathbf{R}^{(r)}} = \mathbf{0}$ gives the stationary point of $\mathcal{Q}(\mathbf{\Theta})$ w.r.t. $\mathbf{R}^{(r)}$:

$$\hat{\mathbf{R}}^{(r)} = \hat{\mathbf{\Psi}}_d^{(r)^{-\frac{1}{2}}} (\frac{1}{n}\sum_{k=1}^{n} \mathbf{G}_k^{(r)^{\mathsf{T}}}) \hat{\mathbf{\Psi}}_d^{(r)^{-\frac{1}{2}}}.$$

Then, combining the stationary point w.r.t. $\mathbf{R}^{(r)}$ across all modalities ($r = 1, \ldots, R$) gives the stationary

point w.r.t. $\mathbf{R}$:

$$\begin{aligned}
\hat{\mathbf{R}} &= \mathsf{diag}(\mathbf{R}^{(1)}, \mathbf{R}^{(2)}, \ldots, \mathbf{R}^{(R)}) \\
&= \mathsf{diag}(\hat{\boldsymbol{\Psi}}_d^{(1)^{-\frac{1}{2}}}(\frac{1}{n}\sum_{k=1}^{n}\mathbf{G}_k^{(1)^{\mathsf{T}}})\hat{\boldsymbol{\Psi}}_d^{(1)^{-\frac{1}{2}}}, \hat{\boldsymbol{\Psi}}_d^{(2)^{-\frac{1}{2}}}(\frac{1}{n}\sum_{k=1}^{n}\mathbf{G}_k^{(2)^{\mathsf{T}}})\hat{\boldsymbol{\Psi}}_d^{(2)^{-\frac{1}{2}}}, \ldots, \hat{\boldsymbol{\Psi}}_d^{(R)^{-\frac{1}{2}}}(\frac{1}{n}\sum_{k=1}^{n}\mathbf{G}_k^{(R)^{\mathsf{T}}})\hat{\boldsymbol{\Psi}}_d^{(R)^{-\frac{1}{2}}}) \\
&= \hat{\boldsymbol{\Psi}}_d^{-\frac{1}{2}}(\mathsf{diag}(\frac{1}{n}\sum_{k=1}^{n}\mathbf{G}_k^{(1)^{\mathsf{T}}}, \frac{1}{n}\sum_{k=1}^{n}\mathbf{G}_k^{(2)^{\mathsf{T}}}, \ldots, \frac{1}{n}\sum_{k=1}^{n}\mathbf{G}_k^{(R)^{\mathsf{T}}}))\hat{\boldsymbol{\Psi}}_d^{-\frac{1}{2}} \\
&= \hat{\boldsymbol{\Psi}}_d^{-\frac{1}{2}}(\frac{1}{n}\sum_{k=1}^{n}\mathsf{diag}(\mathbf{G}_k^{(1)^{\mathsf{T}}}, \mathbf{G}_k^{(2)^{\mathsf{T}}}, \ldots, \mathbf{G}_k^{(R)^{\mathsf{T}}}))\hat{\boldsymbol{\Psi}}_d^{-\frac{1}{2}} \\
&= \hat{\boldsymbol{\Psi}}_d^{-\frac{1}{2}}(\frac{1}{n}\sum_{k=1}^{n}\mathsf{Bdiag}_{\boldsymbol{\Psi}}[\mathbf{G}_k^{\mathsf{T}}])\hat{\boldsymbol{\Psi}}_d^{-\frac{1}{2}}.
\end{aligned}$$

Therefore, the joint MLEs of $(\mathbf{R}, \boldsymbol{\Psi}_d)$ are

$$\begin{cases}
\hat{\boldsymbol{\Psi}}_d = \mathsf{Diag}[\frac{1}{n}\sum_{k=1}^{n}\mathbf{G}_k] = \mathsf{Diag}[\mathbf{S}] \\
\hat{\mathbf{R}} = \hat{\boldsymbol{\Psi}}_d^{-\frac{1}{2}}(\frac{1}{n}\sum_{k=1}^{n}\mathsf{Bdiag}_{\boldsymbol{\Psi}}[\mathbf{G}_k^{\mathsf{T}}])\hat{\boldsymbol{\Psi}}_d^{-\frac{1}{2}}
\end{cases}.$$

Hence,

$$\begin{aligned}
\hat{\mathbf{R}}_{\mathsf{ridge}} &= \lambda\mathsf{Diag}[\mathbf{S}]^{-\frac{1}{2}}(\frac{1}{n}\sum_{k=1}^{n}\mathsf{Bdiag}_{\boldsymbol{\Psi}}[\mathbf{G}_k])\mathsf{Diag}[\mathbf{S}]^{-\frac{1}{2}} + (1-\lambda)\mathbf{I} \\
&= \lambda\hat{\boldsymbol{\Psi}}_d^{-\frac{1}{2}}(\frac{1}{n}\sum_{k=1}^{n}\mathsf{Bdiag}_{\boldsymbol{\Psi}}[\mathbf{G}_k^{\mathsf{T}}])\hat{\boldsymbol{\Psi}}_d^{-\frac{1}{2}} + (1-\lambda)\mathbf{I} \\
&= \lambda\hat{\mathbf{R}} + (1-\lambda)\mathbf{I}
\end{aligned}$$

In addition, the ridge estimator of the covariance matrix $\boldsymbol{\Psi}$ is derived as follows.

$$\begin{aligned}
\hat{\boldsymbol{\Psi}}_{\mathsf{ridge}} &= \hat{\boldsymbol{\Psi}}_{d,\mathsf{ridge}}^{\frac{1}{2}}\hat{\mathbf{R}}_{\mathsf{ridge}}\hat{\boldsymbol{\Psi}}_{d,\mathsf{ridge}}^{\frac{1}{2}} \\
&= \lambda^{-\frac{1}{2}}\mathsf{Diag}[\mathbf{S}]^{\frac{1}{2}}[\lambda\hat{\mathbf{R}} + (1-\lambda)\mathbf{I}]\lambda^{-\frac{1}{2}}\mathsf{Diag}[\mathbf{S}]^{\frac{1}{2}} \\
&= \mathsf{Diag}[\mathbf{S}]^{\frac{1}{2}}\hat{\mathbf{R}}\mathsf{Diag}[\mathbf{S}]^{\frac{1}{2}} + \frac{1-\lambda}{\lambda}\mathsf{Diag}[\mathbf{S}]^{\frac{1}{2}}\mathsf{Diag}[\mathbf{S}]^{\frac{1}{2}} \\
&= \hat{\boldsymbol{\Psi}}_d^{\frac{1}{2}}\hat{\boldsymbol{\Psi}}_d^{-\frac{1}{2}}(\frac{1}{n}\sum_{k=1}^{n}\mathsf{Bdiag}_{\boldsymbol{\Psi}}[\mathbf{G}_k^{\mathsf{T}}])\hat{\boldsymbol{\Psi}}_d^{-\frac{1}{2}}\hat{\boldsymbol{\Psi}}_d^{\frac{1}{2}} + (\frac{1}{\lambda}-1)\hat{\boldsymbol{\Psi}}_d \\
&= \frac{1}{n}\sum_{k=1}^{n}\mathsf{Bdiag}_{\boldsymbol{\Psi}}[\mathbf{G}_k^{\mathsf{T}}] + (\frac{1}{\lambda}-1)\hat{\boldsymbol{\Psi}}_d \\
&= \hat{\boldsymbol{\Psi}} + (\frac{1}{\lambda}-1)\hat{\boldsymbol{\Psi}}_d,
\end{aligned}$$

where $\lambda \in (0,1)$.

1.3 Comparison of Missing Data Imputation Methods

SNF, NEMO, and DGCCA do not natively support the handling of missing values, missing data imputation is a necessary preprocessing step before they can be used for multi-modal data integration. Since none of these methods provide specific recommendations for handling missing data in their package documentation or tutorials, we applied k-nearest neighbors (kNN) imputation with k=5 consistently across all simulation studies and real data applications to ensure a fair and standardized comparison. To further investigate how these methods' performance may be impacted by imputation strategies or hyperparameter choices, we have carried out a more comprehensive study of missing imputations based on the simulated datasets. We include random imputation as a baseline method, where missing values are replaced with randomly sampled values from a Gaussian distribution inferred for the same variable. In addition, we also consider kNN imputation, iterative EM-PCA imputation, and iterative model-based imputation. For the kNN approach, we assess performance across different numbers of neighbors, specifically $k = 5, 10, 15, 20$. The ARI and NMI scores of SNF combined with different imputation methods in comparison with GPCCA (regularization parameter $\lambda = 2/3$) are presented in Supplementary Figures S18-19, respectively. These results indicate that GPCCA consistently outperforms SNF regardless of the imputation strategy applied. Moreover, no single imputation method yields consistently superior performance for SNF across all settings. Importantly, we caution against selecting imputation methods based on these comparisons, as metrics such as ARI and NMI require ground truth labels, which are typically unavailable in real-world applications. For NEMO (Supplementary Figures S20-21) and DGCCA (Supplementary Figures S22-23), although the specific ARI and NMI scores vary, the overall trends are consistent with those observed for SNF.

From Figure S18-S19 for SNF, Figure S20-S21 for NEMO and Figure S22-S23 for DGCCA, both heatmaps of ARIs and NMIs indicate that, in general, there is no best missing imputation technique. Also, the impact of using different imputation techniques on final clustering performance is not significant, except for Random Imputation and Iterative EM-PCA Imputation. Additionally, for kNN imputation, under different k values ($k = 5, 10, 15, 20$), the ARIs and NMIs are fairly close. Therefore, it is a reasonable choice to use kNN imputation with $k = 5$, which is the default of R function `VIM::kNN()` and also computationally the fastest.

1.4 Implementation Details of DGCCA

In alignment with the original DGCCA paper, which adopts a fixed network depth and size across modalities, we design a general training pipeline to apply DGCCA consistently across all simulation studies and real data applications. For each modality, we use an identical fully connected neural network architecture comprising three hidden layers with 512, 256, and 128 units, respectively. The output dimensionality is fixed at 64. In addition, we set the learning rate to 0.001 to ensure stable convergence while exploring local optima, and the number of training epochs to 100 to allow sufficient convergence without overfitting. The batch size is adjusted according to dataset size: for smaller datasets (e.g.,

simulation studies and TCGA multi-omics data), we use a batch size of 25, while for larger datasets (e.g., multi-view image data and DLPFC data), we use a batch size of 100. Since DGCCA does not support missing data, we apply k-nearest neighbors (kNN) imputation with $k = 5$ as a preprocessing step whenever a dataset contains missing values.

1.5 Features in the Multi-view Image data

The description of all four feature extraction methods used in the multi-feature image dataset is provided as follows.

Modality 1: The Fourier coefficients of character shapes represent a digit's image in the frequency domain by computing its Fourier transform. This approach effectively captures global shape features rather than localized details, making it particularly useful in applications where the overall outline and structure are more important than texture.

Modality 2: Profile correlations are obtained by calculating the correlations between the intensity profiles of each digit image along specific directions. These correlations capture the continuity of the shape along the chosen axis and providing insights into the structural features of the digits.

Modality 3: Karhunen-Loève coefficients, also known as principal component coefficients, are obtained through the Karhunen-Loève transform, which decomposes an image into orthogonal components that capture the most significant variance in the data. These coefficients are widely used in image and pattern recognition for efficient feature extraction, particularly in high-dimensional data where dimensionality reduction and concise representation are crucial.

Modality 4: Zernike moments are a set of orthogonal complex polynomials that capture shape features in an image by combining radial and angular functions. They are particularly effective at capturing rotationally invariant features, making them useful in applications where rotation or scaling occurs.

1.6 Processing of Multi-Omics Data

We process the TCGA multi-omics data for each cancer type as follows before applying the multi-modal methods to analyze them.

- For the gene expression modality, we only retain genes whose average expression levels are at least 10. For DNA methylation and miRNA expression, only features with less than 50% missing rates are retained.

- Within each modality, we sort features based on their empirical variance. For gene expression and DNA methylation, we retain the top 5000 and 10,000 features, respectively. All features in the miRNA expression modality are retained.

- To further screen for informative features within each modality, we follow these steps. (1) Compute the sample correlation matrix. (2) Define a threshold for the maximum absolute correlation, set as the 99th percentile of absolute correlations. (3) Starting with the variable with the smallest

variability, check its absolute correlation with all other features. If any correlation exceeds the pre-defined threshold, remove the current variable. Repeat this process for the next variable. The target dimensions for the three modalities are $m_1 = 1000$, $m_2 = 2000$ and $m_3 = 500$. If the feature removal process stops and the dimension of modality is larger than our target, we keep the top features based on feature variability. However, if the modality's dimension approaches the target, we stop the deletion process to ensure it does not drop below the desired size.

2 Supplementary Table

Table S1: A summary of missing data imputation methods used in method evaluations.

Abbreviation	Imputation method	R Package	Function
Random	Random imputation	-	-
impPCA	Iterative EM-PCA imputation	`VIM`	`impPCA`
IRMI	Iterative robust model-based imputation	`VIM`	`irmi`
kNN ($k = 5$)	k-Nearest neighbor imputation	`VIM`	`kNN`
kNN ($k = 10$)	k-Nearest neighbor imputation	`VIM`	`kNN`
kNN ($k = 15$)	k-Nearest neighbor imputation	`VIM`	`kNN`
kNN ($k = 20$)	k-Nearest neighbor imputation	`VIM`	`kNN`

Table S2: Sample size of each modality and phenotype data for all 10 TCGA cancers after preprocessing.

Cancer type	Gene expression	DNA methylation	miRNA expression	Phenotype
LUAD	$n = 460$	$n = 412$	$n = 402$	$n = 464$
COAD	$n = 256$	$n = 267$	$n = 223$	$n = 275$
LUSC	$n = 429$	$n = 309$	$n = 284$	$n = 429$
SARC	$n = 251$	$n = 253$	$n = 249$	$n = 253$
BLCA	$n = 383$	$n = 387$	$n = 385$	$n = 387$
BRCA	$n = 1059$	$n = 761$	$n = 721$	$n = 1061$
HNSC	$n = 492$	$n = 500$	$n = 456$	$n = 500$
KIRC	$n = 517$	$n = 311$	$n = 233$	$n = 518$
LGG	$n = 486$	$n = 486$	$n = 482$	$n = 486$
LIHC	$n = 352$	$n = 357$	$n = 350$	$n = 357$

Table S3: NMI scores of clustering results using different analytical methods. PPCA-all, GPCCA, MOFA, SNF, NEMO and DGCCA are applied to the multi-modality data, whereas PPCA-fou, PPCA-fac, PPCA-kar, and PPCA-zer represent PPCA applied to Fourier coefficients, Profile correlations, Karhunen-Loève coefficients, and Zernike moments, respectively. For each dataset, the highest NMI value is highlighted in bold.

Methods	Complete	MCAR 20%	MCAR 40%	MNAR 25%	MNAR 50%
PPCA-fou	0.6921	0.7151	0.6447	0.6828	0.7409
PPCA-fac	0.7573	0.7510	0.7570	0.7438	0.7318
PPCA-kar	0.5578	0.6375	0.6816	0.2937	0.3019
PPCA-zer	0.7168	0.7040	0.6205	0.7113	0.7086
PPCA-all	0.7813	0.7850	0.8009	0.7501	0.6896
GPCCA	**0.8696**	**0.9054**	0.8380	**0.8941**	**0.8993**
MOFA	0.8478	0.8426	**0.8445**	0.8629	0.8418
SNF	0.8356	0.8015	0.8077	0.8324	0.7988
NEMO	0.8169	0.8117	0.7586	0.7522	0.7569
DGCCA	0.7632	0.7757	0.7762	0.7450	0.7637

Table S4: Comparison between informative and backgrounds gene sets across 10 TCGA cancer types based on MOFA's results. "Total # of Genes in OS" represents the overlap between genes included in the OncoSearch database and those used in the multi-modal analysis. For each background and informative gene set associated with a specific cancer type, the "% of genes in OS among all available" is calculated as the number of overlapping genes divided by the total number of genes in OS, while the "% of genes in OS among all reported" is the number of overlapping genes divided by the total number of genes in the given gene set.

		Background gene set		Informative gene set	
Cancer type	Total # of genes in OS	# (or %) of genes in OS among all available	% of genes in OS among all reported	# (or %) of genes in OS among all available	% of genes in OS among all reported
LUAD	30	4 (13.33%)	1.56%	15 (50.00%)	4.45%
COAD	91	13 (14.29%)	6.31%	25 (27.47%)	12.82%
LUSC	4	2 (50.00%)	0.90%	0 (0.00%)	0.00%
SARC	81	14 (17.28%)	6.09%	37 (45.68%)	7.49%
BLCA	43	12 (27.91%)	4.82%	20 (46.51%)	5.95%
BRCA	113	11 (9.73%)	5.76%	65 (57.52%)	15.40%
HNSC	21	5 (23.81%)	1.92%	5 (23.81%)	2.63%
KIRC	4	1 (25.00%)	0.39%	2 (50.00%)	0.49%
LGG	100	21 (21.00%)	8.50%	37 (37.00%)	11.38%
LIHC	107	22 (20.56%)	8.00%	48 (44.86%)	17.91%

Table S5: Comparison between informative and backgrounds gene sets across 10 TCGA cancer types based on PPCA's results. "Total # of Genes in OS" represents the overlap between genes included in the OncoSearch database and those used in the multi-modal analysis. For each background and informative gene set associated with a specific cancer type, the "% of genes in OS among all available" is calculated as the number of overlapping genes divided by the total number of genes in OS, while the "% of genes in OS among all reported" is the number of overlapping genes divided by the total number of genes in the given gene set.

		Background gene set		Informative gene set	
Cancer type	Total # of genes in OS	# (or %) of genes in OS among all available	% of genes in OS among all reported	# (or %) of genes in OS among all available	% of genes in OS among all reported
LUAD	30	12 (40.00%)	1.89%	14 (46.67%)	5.45%
COAD	91	50 (54.95%)	7.66%	14 (15.38%)	14.00%
LUSC	4	3 (75.00%)	0.57%	1 (25.00%)	0.38%
SARC	81	55 (67.90%)	8.41%	14 (17.28%)	7.49%
BLCA	43	15 (34.88%)	2.85%	14 (32.56%)	7.37%
BRCA	113	46 (40.71%)	9.11%	24 (21.24%)	13.33%
HNSC	21	10 (47.62%)	1.75%	8 (38.10%)	3.10%
KIRC	4	1 (25.00%)	0.16%	2 (50.00%)	0.79%
LGG	100	34 (34.00%)	8.35%	33 (33.00%)	12.36%
LIHC	107	49 (45.79%)	8.93%	41 (38.32%)	16.27%

Table S6: Comparison of clustering performance (ARI) on the DLPFC dataset. PPCA-all, GPCCA, MOFA, SNF, NEMO and DGCCA are applied to the multi-modal data; PPCA-expr represents PPCA applied to the gene expression data alone; PPCA-img represents PPCA applied to image features alone. For each slice (denoted by the corresponding ID), the highest ARI value is highlighted in bold.

Methods	151673	151674	151675	151676
PPCA-expr	0.1272	0.1612	0.1768	0.1338
PPCA-img	0.0539	0.0940	0.0871	0.0962
PPCA-all	0.1350	0.1577	0.1493	0.1510
GPCCA	0.1816	**0.2262**	**0.2258**	0.2366
MOFA	0.1853	0.1903	0.1831	**0.2487**
SNF	0.0714	0.0884	0.1056	0.1228
NEMO	**0.1934**	0.1540	0.1629	0.1978
DGCCA	0.1334	0.1329	0.1265	0.1458

Table S7: Comparison of clustering performance (NMI) on the DLPFC dataset. PPCA-all, GPCCA, MOFA, SNF, NEMO and DGCCA are applied to the multi-modal data; PPCA-expr represents PPCA applied to the gene expression data alone; PPCA-img represents PPCA applied to image features alone. For each slice (denoted by the corresponding ID), the highest NMI value is highlighted in bold.

Methods	151673	151674	151675	151676
PPCA-expr	0.2544	0.2372	0.3078	0.2445
PPCA-img	0.1194	0.1489	0.1669	0.1579
PPCA-all	0.2418	0.2343	0.2986	0.2559
GPCCA	0.3131	**0.3300**	**0.3386**	0.3331
MOFA	**0.3135**	0.3062	0.3217	**0.3689**
SNF	0.1415	0.1567	0.1720	0.1755
NEMO	0.2597	0.1712	0.2359	0.2554
DGCCA	0.2235	0.2357	0.2214	0.2387

3 Supplementary Figures

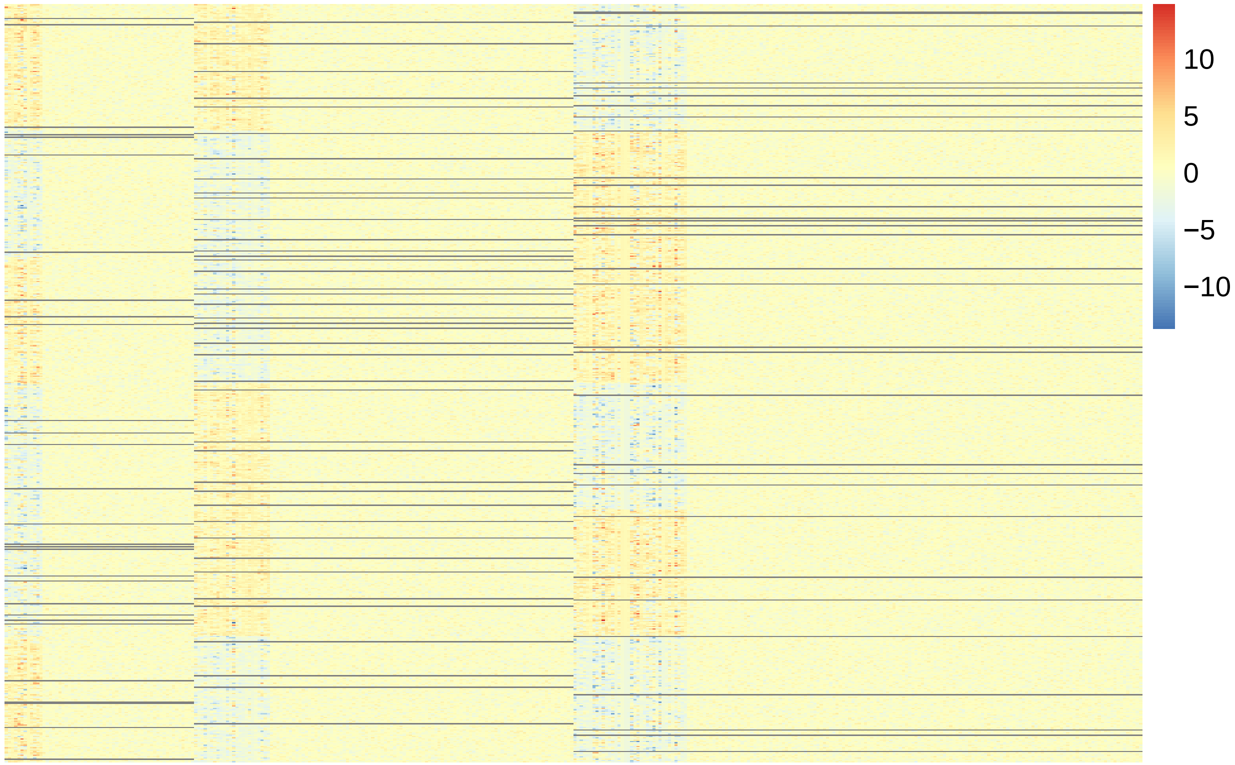

Figure S1: Heatmap of synthetic data in Case C with $p = 0.1$ and $\rho = 0.5$. The columns correspond to features and the rows correspond to subjects. Grey bars denote samples with missing modalities.

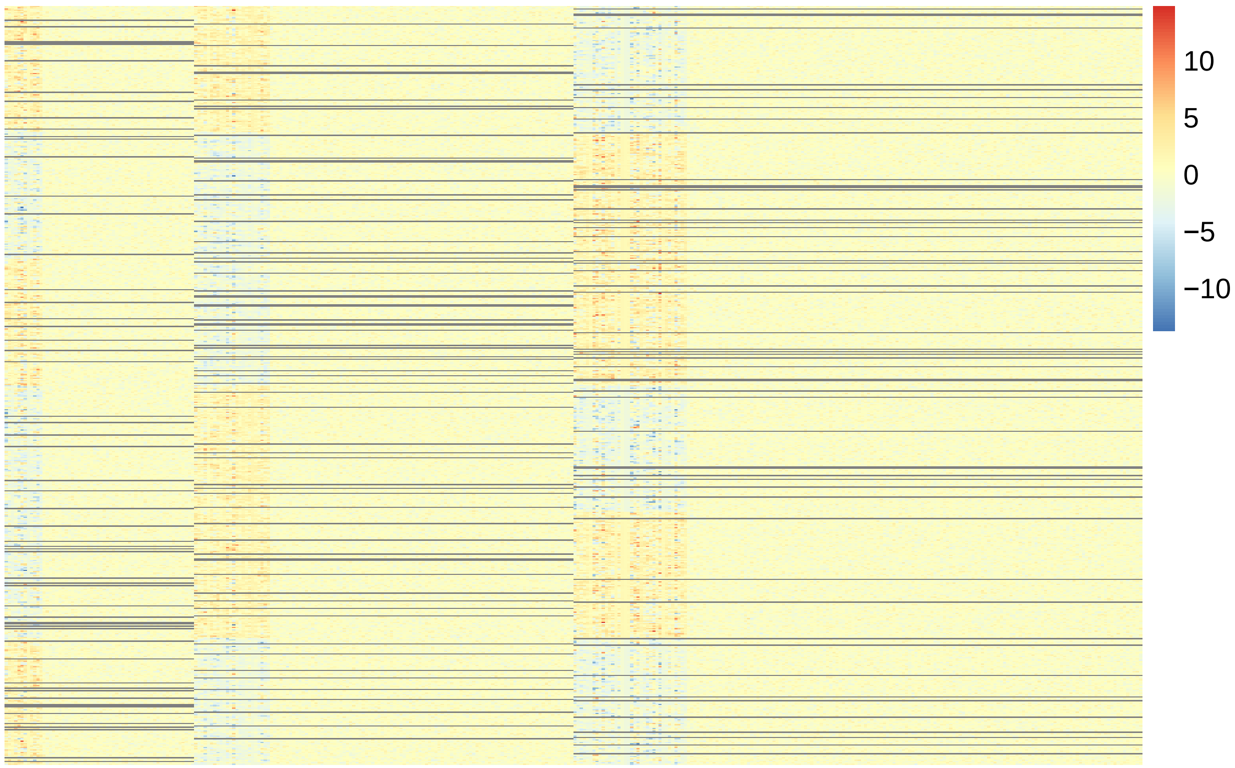

Figure S2: Heatmap of synthetic data in Case C with $p = 0.2$ and $\rho = 0.5$. The columns correspond to features and the rows correspond to subjects. Grey bars denote samples with missing modalities.

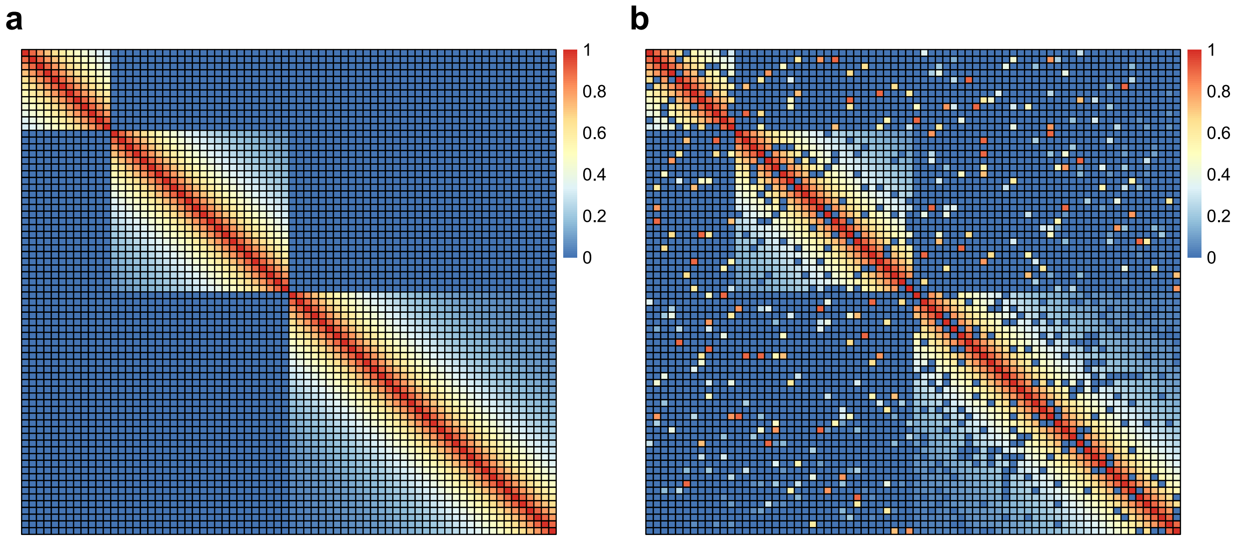

Figure S3: Example covariance matrices of simulated data. **a.** True correlation matrix for data generation of informative features in Case A with $\rho = 0.9$. **b.** True correlation matrix for data generation of informative features in Case D with $\rho = 0.9$, which is obtained by swapping $1/6$ within-modality correlation entries with between-modality zero entries.

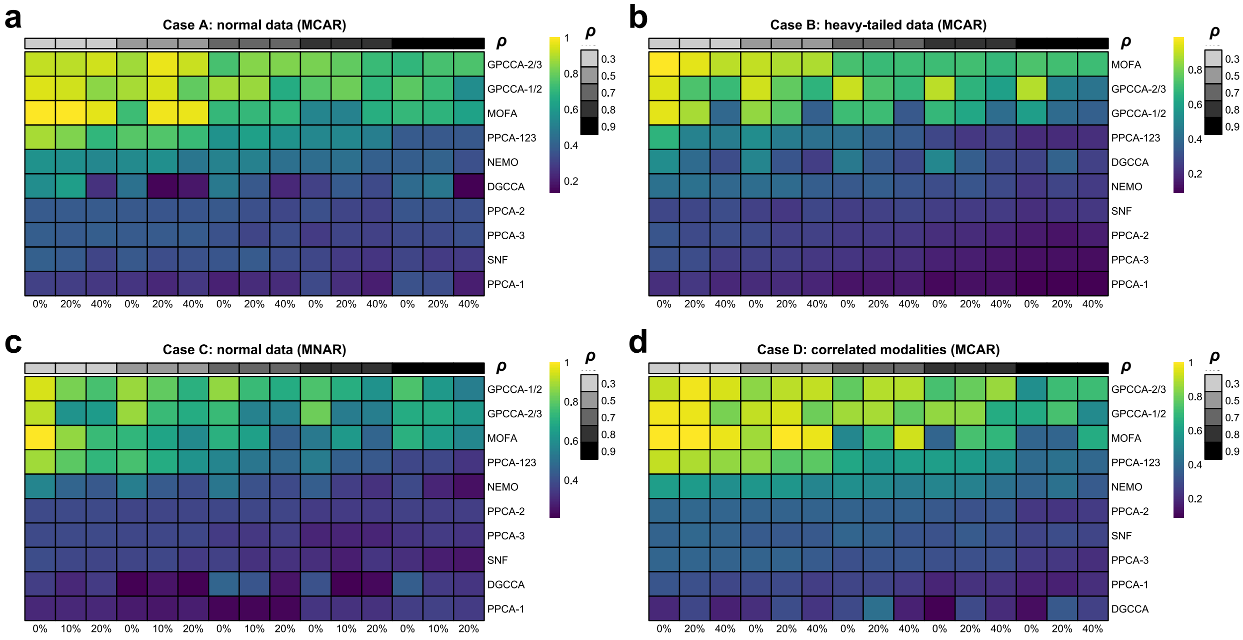

Figure S4: Comparison of clustering performance (NMI) in simulation study. **a.** Case A: normal data (MCAR). **b.** Case B: heavy-tailed data (MCAR). **c.** Case C: normal data (MNAR). **d.** Case D: correlated modalities (MCAR). GPCCA with different regularization parameters are denoted as GPCCA-2/3 and GPCCA-1/2. PPCA applied to concatenated modalities is denoted as PPCA-123. PPCA applied to individual modalities is denoted as PPCA-1, PPCA-2, and PPCA-3, respectively. Methods are ordered from high to low, based on their average NMI across all scenarios with different missing levels and correlation levels. In Cases A, B, and D, the horizontal labels on the bottom of the heatmap represent missing rates; in Case C, the horizontal labels represent the baseline probability (p) of modality-wise missingness (see Methods).

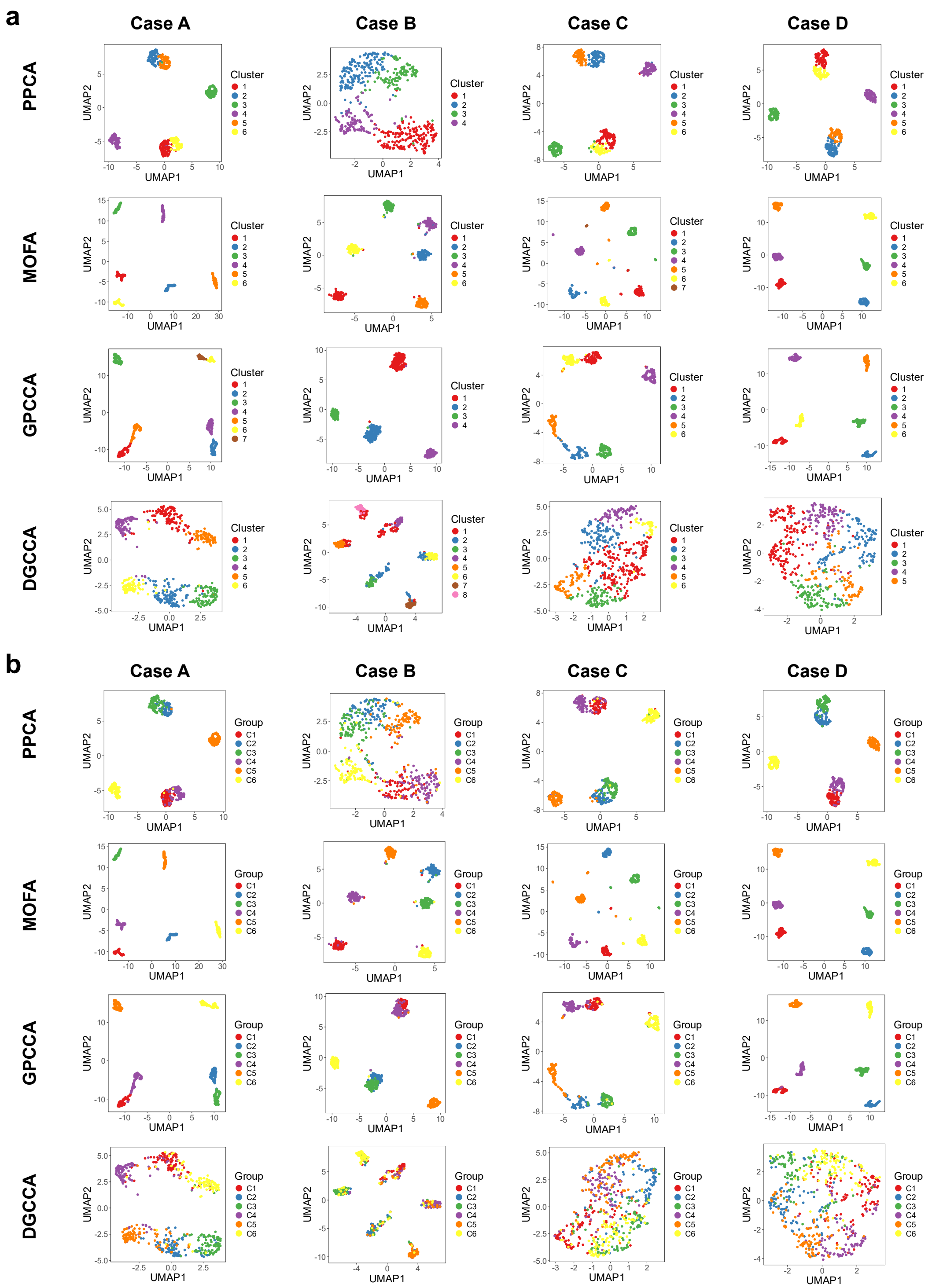

Figure S5: UMAP projections based on multi-modality analysis by PPCA, MOFA, and GPCCA. **a.** Samples are colored by the inferred clusters. **b.** Samples are colored by the true group labels. The parameter setting for each case: Case A (20% missing rate, $\rho = 0.3$), Case B (20% missing rate, $\rho = 0.3$), Case C (10% modality missingness, $\rho = 0.3$), Case D (20% missing rate, $\rho = 0.3$). For GPCCA results, the model with better ARI score is used for visualization: $\lambda = 2/3$ for Cases A, B and D and $\lambda = 1/2$ for Case C.

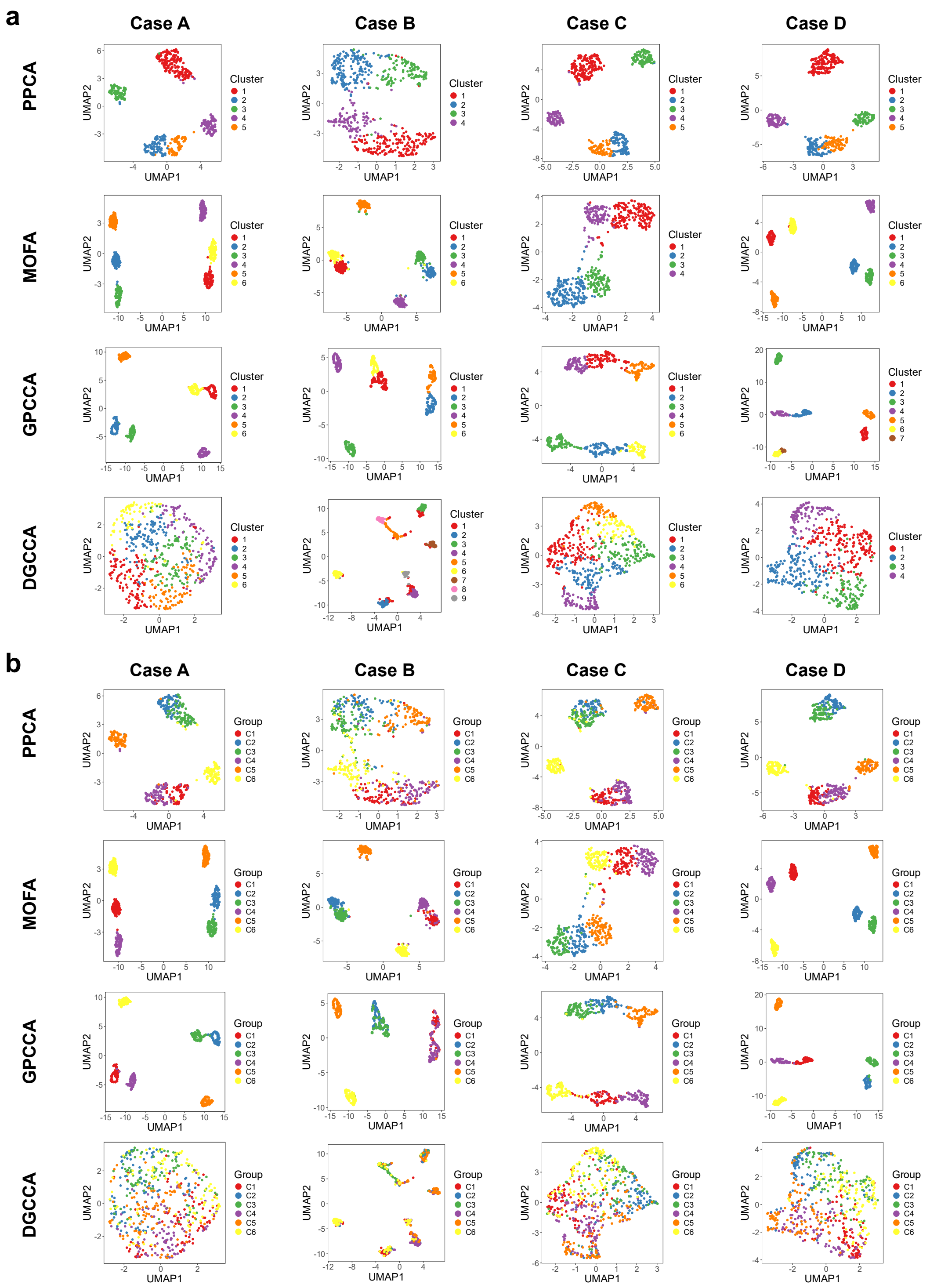

Figure S6: UMAP projections based on multi-modality analysis by PPCA, MOFA, and GPCCA. **a.** Samples are colored by the inferred clusters. **b.** Samples are colored by the true group labels. The parameter setting for each case: Case A (20% missing rate, $\rho = 0.5$), Case B (20% missing rate, $\rho = 0.5$), Case C (10% modality missingness, $\rho = 0.5$), Case D (20% missing rate, $\rho = 0.5$). For GPCCA results, the model with better ARI score is used for visualization: $\lambda = 2/3$ for Cases A, B and D and $\lambda = 1/2$ for Case C.

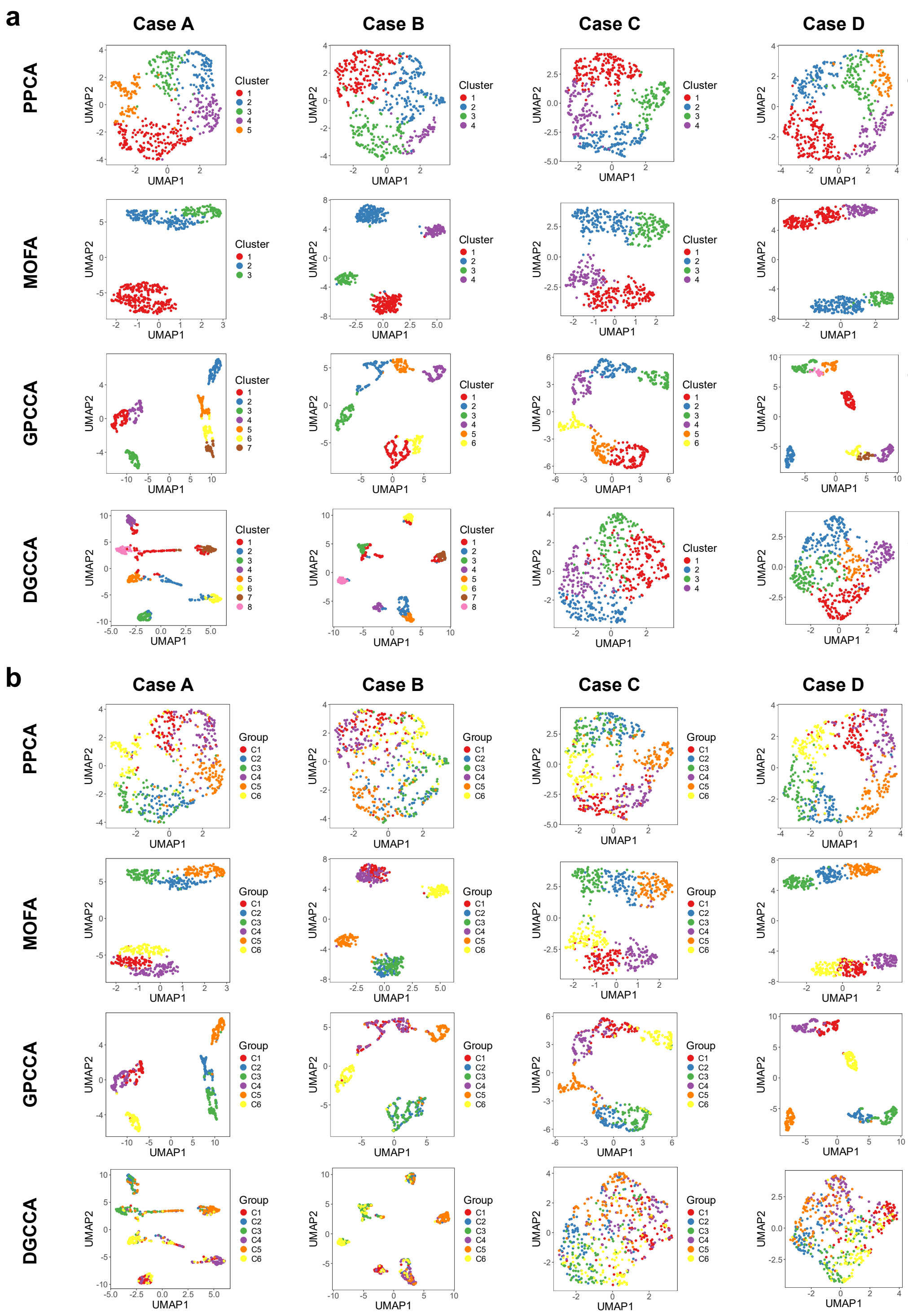

Figure S7: UMAP projections based on multi-modality analysis by PPCA, MOFA, and GPCCA. **a.** Samples are colored by the inferred clusters. **b.** Samples are colored by the true group labels. The parameter setting for each case: Case A (20% missing rate, $\rho = 0.8$), Case B (20% missing rate, $\rho = 0.8$), Case C (10% modality missingness, $\rho = 0.8$), Case D (20% missing rate, $\rho = 0.8$). For GPCCA results, the model with better ARI score is used for visualization: $\lambda = 2/3$ for Cases A, B and D and $\lambda = 1/2$ for Case C.

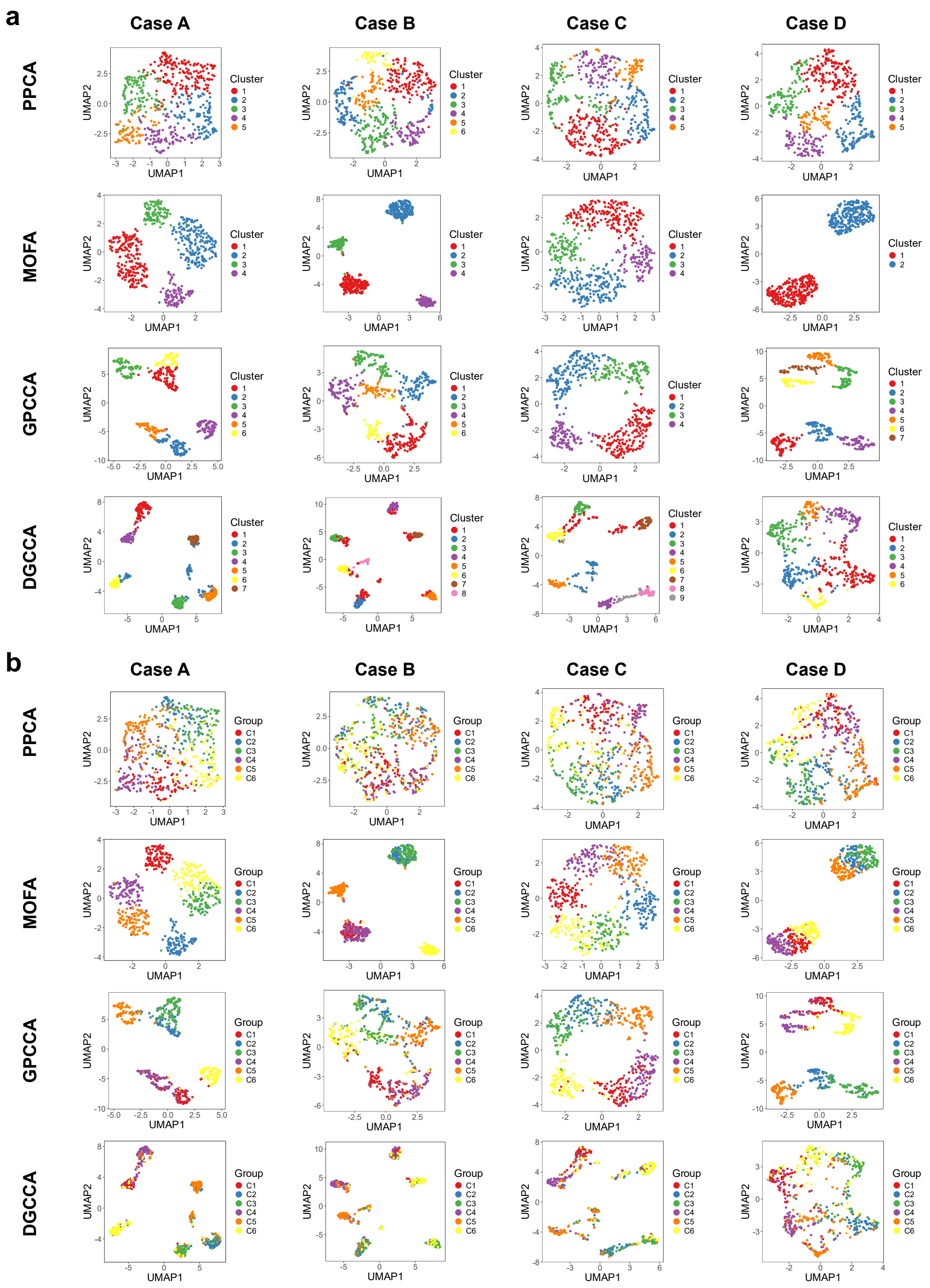

Figure S8: UMAP projections based on multi-modality analysis by PPCA, MOFA, and GPCCA. **a.** Samples are colored by the inferred clusters. **b.** Samples are colored by the true group labels. The parameter setting for each case: Case A (20% missing rate, $\rho = 0.9$), Case B (20% missing rate, $\rho = 0.9$), Case C (10% modality missingness, $\rho = 0.9$), Case D (20% missing rate, $\rho = 0.9$). For GPCCA results, the model with better ARI score is used for visualization: $\lambda = 2/3$ for Case A, B and D and $\lambda = 1/2$ for Case C.

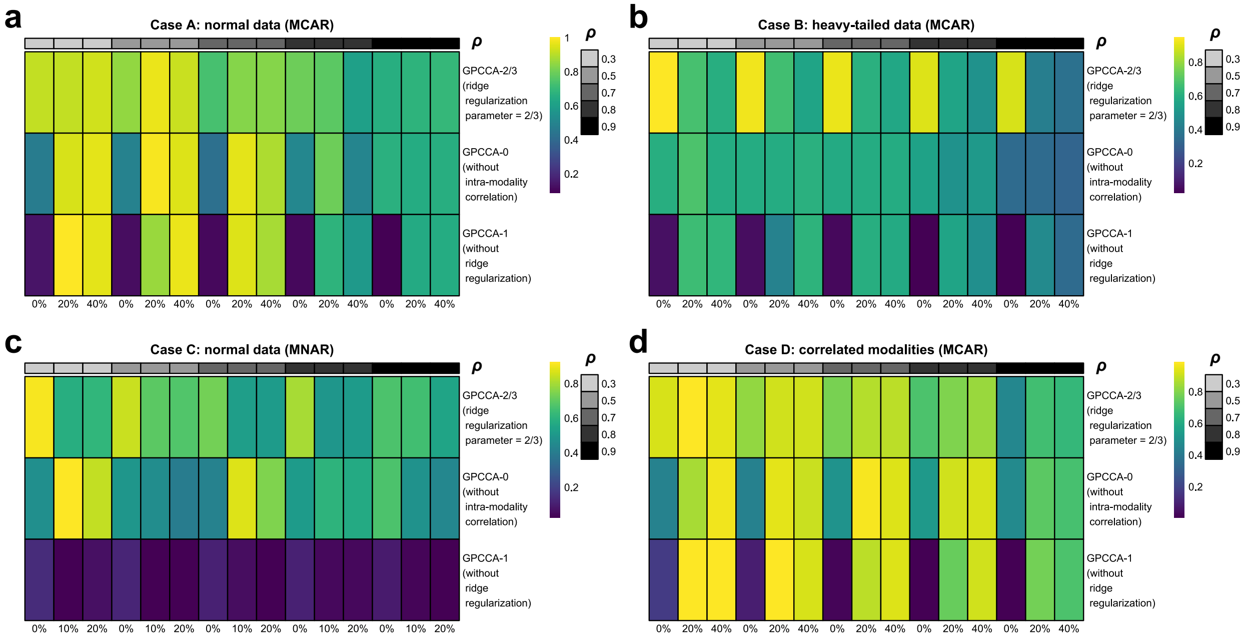

Figure S9: Comparison of clustering performance (ARI) of GPCCA models on simulated datasets. Methods are ordered from highest to lowest based on their average ARI across all scenarios with different missing levels and correlation levels.

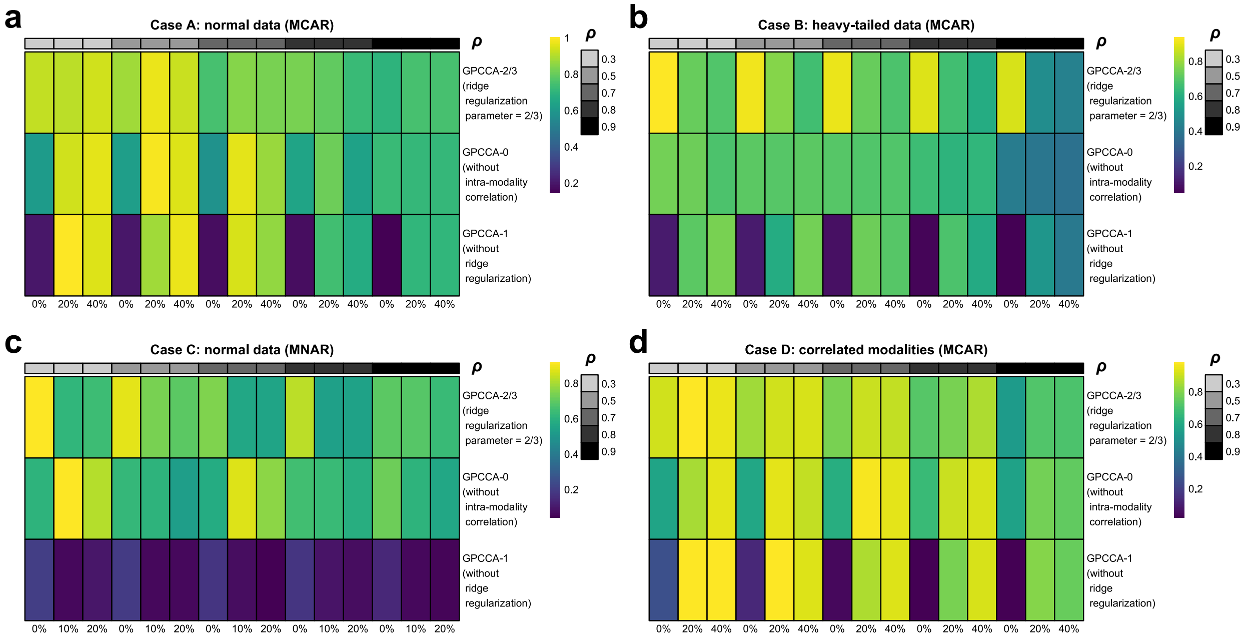

Figure S10: Comparison of clustering performance (NMI) of GPCCA models on simulated datasets. Methods are ordered from highest to lowest based on their average NMI across all scenarios with different missing levels and correlation levels.

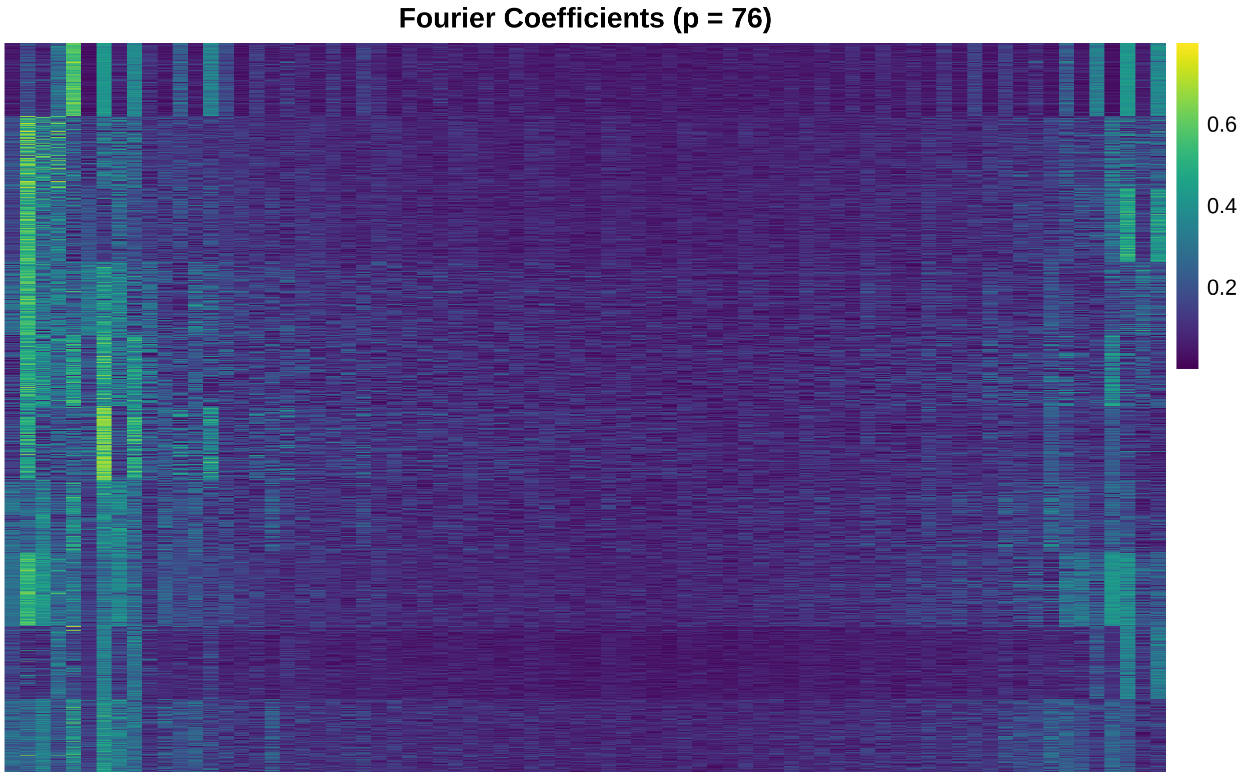

Figure S11: Heatmap of Modality 1 (Fourier coefficients) in the multi-view image data. The columns correspond to features and the rows correspond to samples. The first 200 samples are of class ‘0’, followed by sets of 200 samples for each of the classes in ‘1’ - ‘9’.

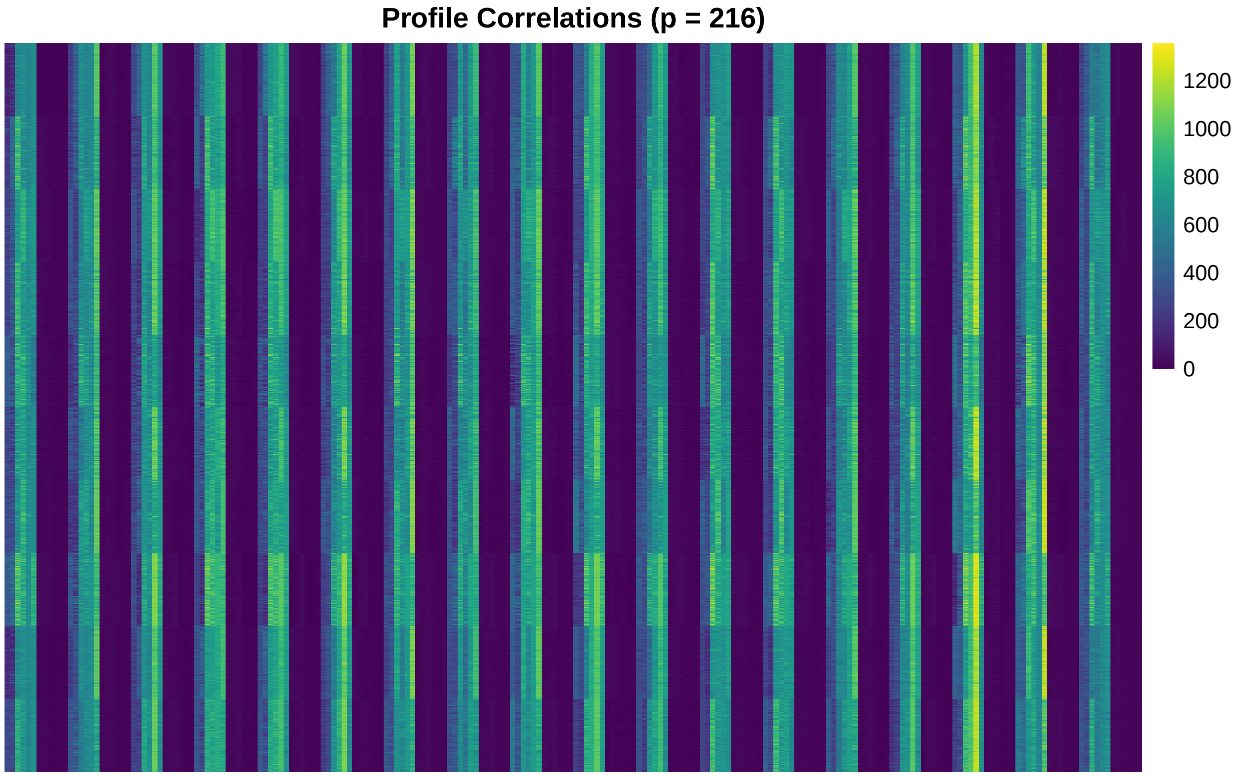

Figure S12: Heatmap of Modality 2 (profile correlation) in the multi-view image data. The columns correspond to features and the rows correspond to samples. The first 200 samples are of class ‘0’, followed by sets of 200 samples for each of the classes in ‘1’ - ‘9’.

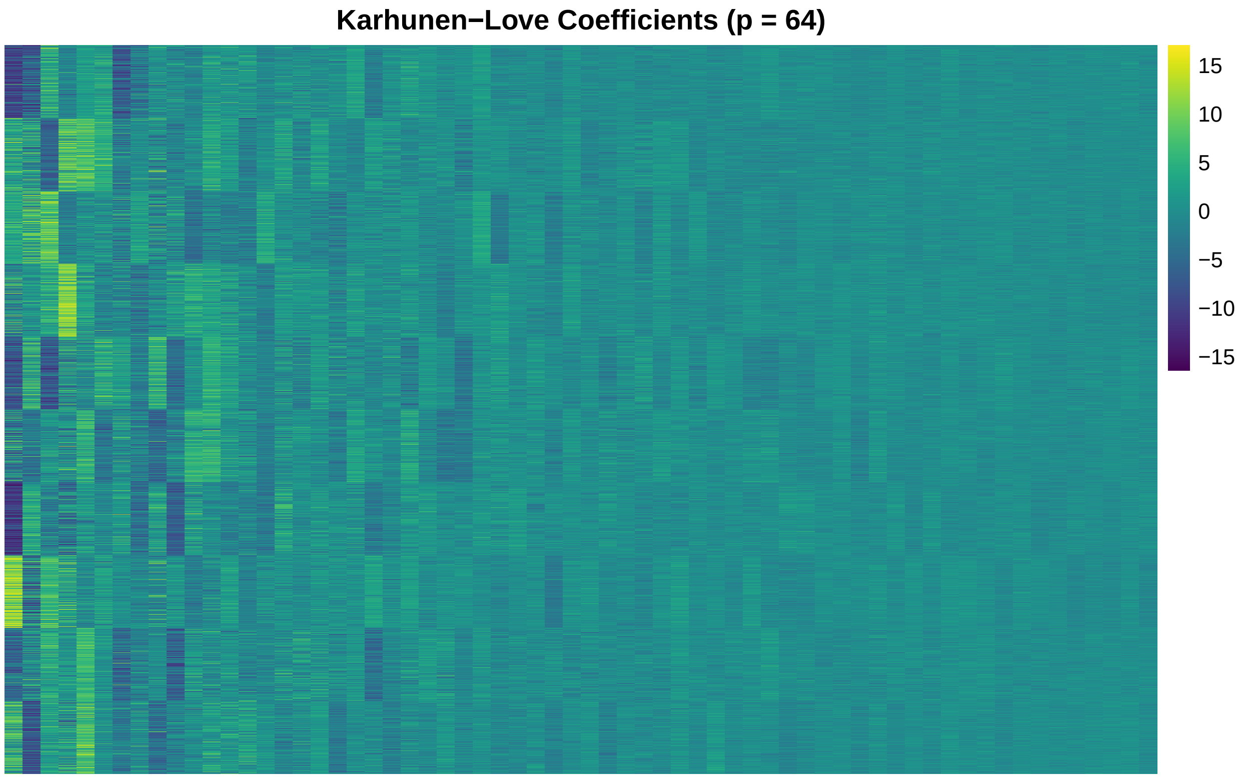

Figure S13: Heatmap of Modality 3 (Karhunen-Love coefficients) in the multi-view image data. The columns correspond to features and the rows correspond to samples. The first 200 samples are of class '0', followed by sets of 200 samples for each of the classes in '1' - '9'.

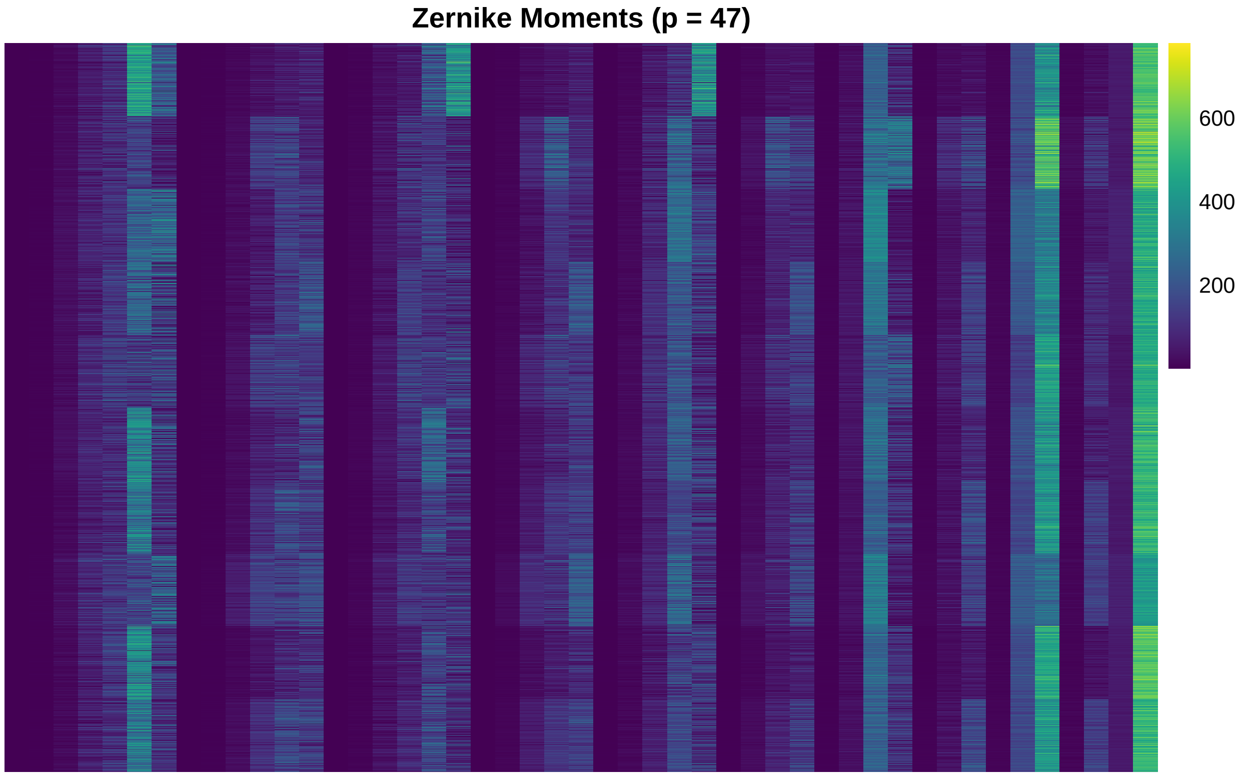

Figure S14: Heatmap of Modality 4 (Zernike moments) in the multi-view image data. The columns correspond to features and the rows correspond to samples. The first 200 samples are of class ‘0’, followed by sets of 200 samples for each of the classes ‘1’ - ‘9’.

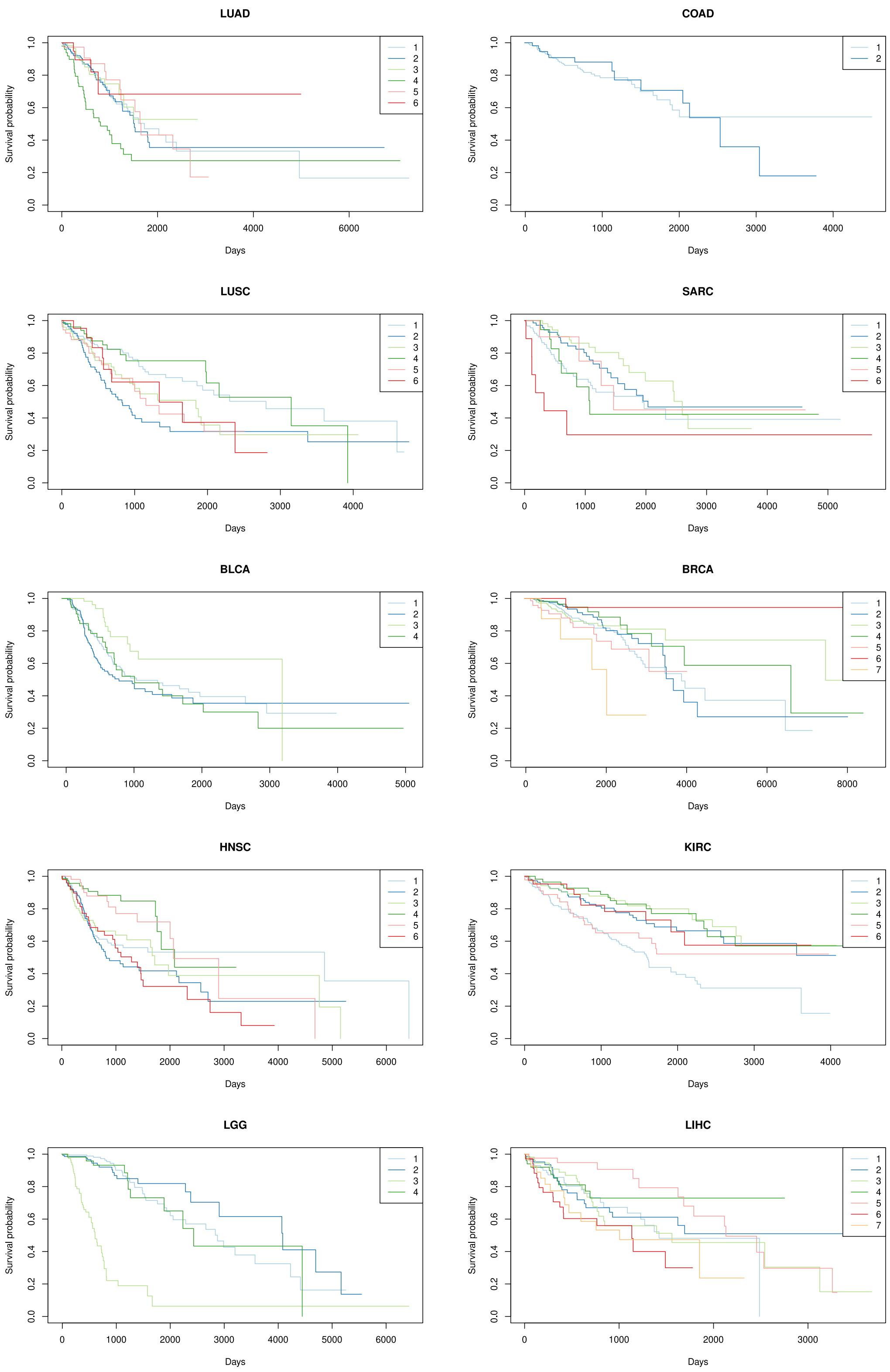

Figure S15: Kaplan–Meier curves comparing the survival time of subjects across identified clusters by GPCCA. The clusters are obtained by applying the Louvain clustering algorithm to the learned subject embeddings of the multi-modal datasets.

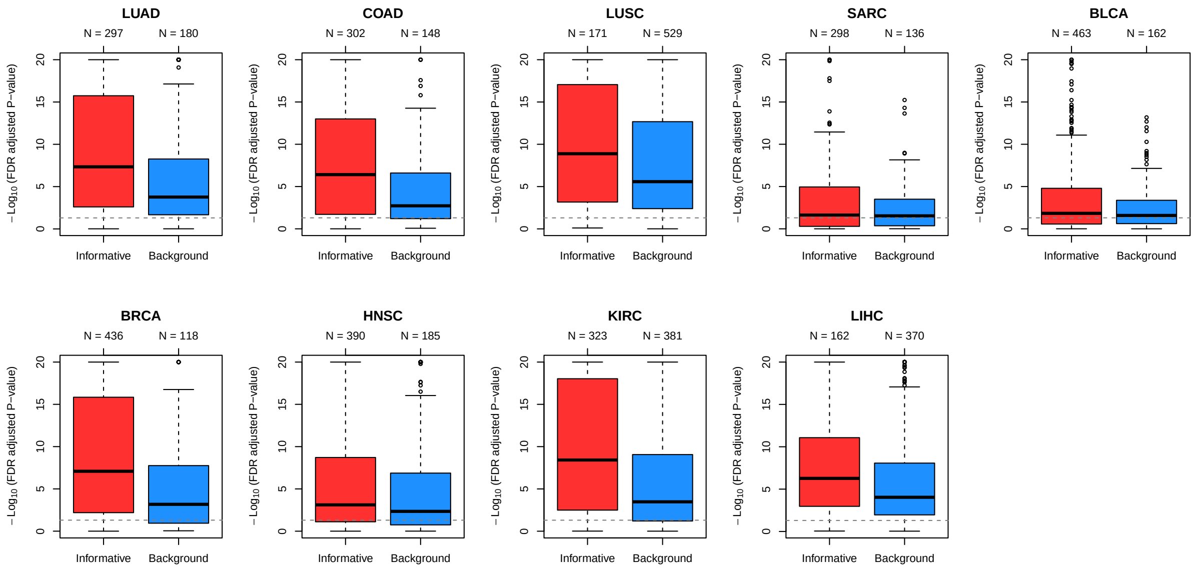

Figure S16: Comparison of the significance between informative background gene sets in differential gene expression (DGE) analysis. The DGE results are retrieved from the OncoDB database. The numbers of genes used to create the boxplot are marked on top of each figure. The gray dashed line denotes the threshold of statistical significance which is 0.05. Low grade glioma (LGG) is excluded because its results are not available from OncoDB.

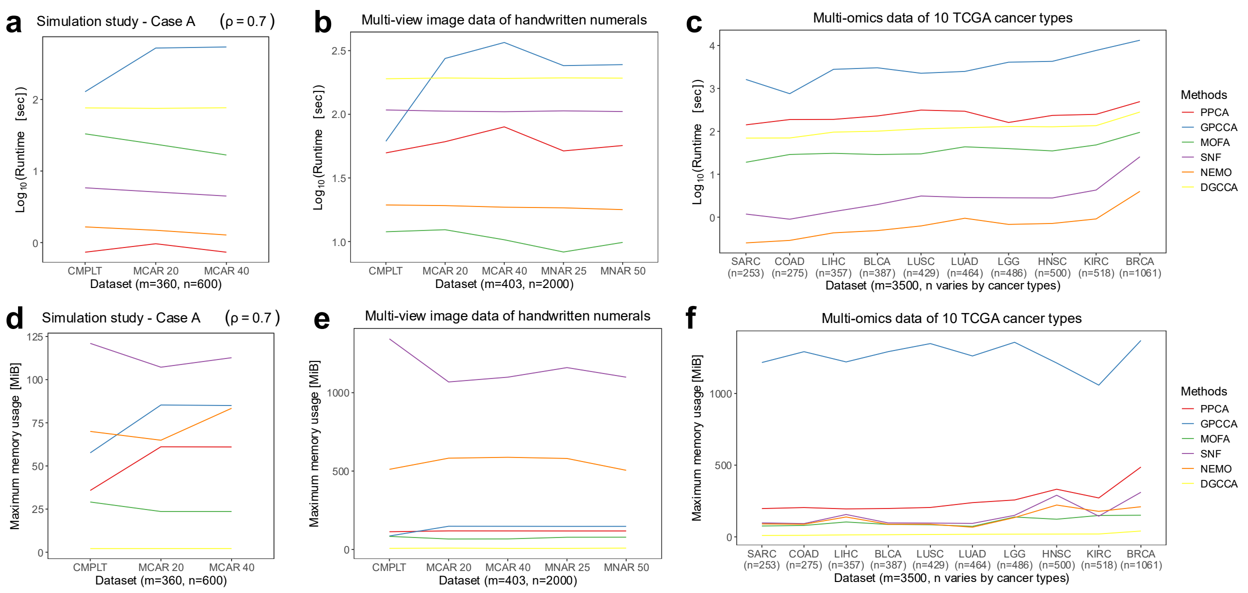

Figure S17: Comparison of computational efficiency. **a**-**c** Running time of the six methods in case A of simulation study (**a**), application of multi-view image data (**b**), and application of multi-omics data (**c**). **d**-**f** Maximum memory usage of the six methods in case A of simulation study (**d**), application of multi-view image data (**e**), and application of multi-omics data (**f**). CMPLT standards for complete data.

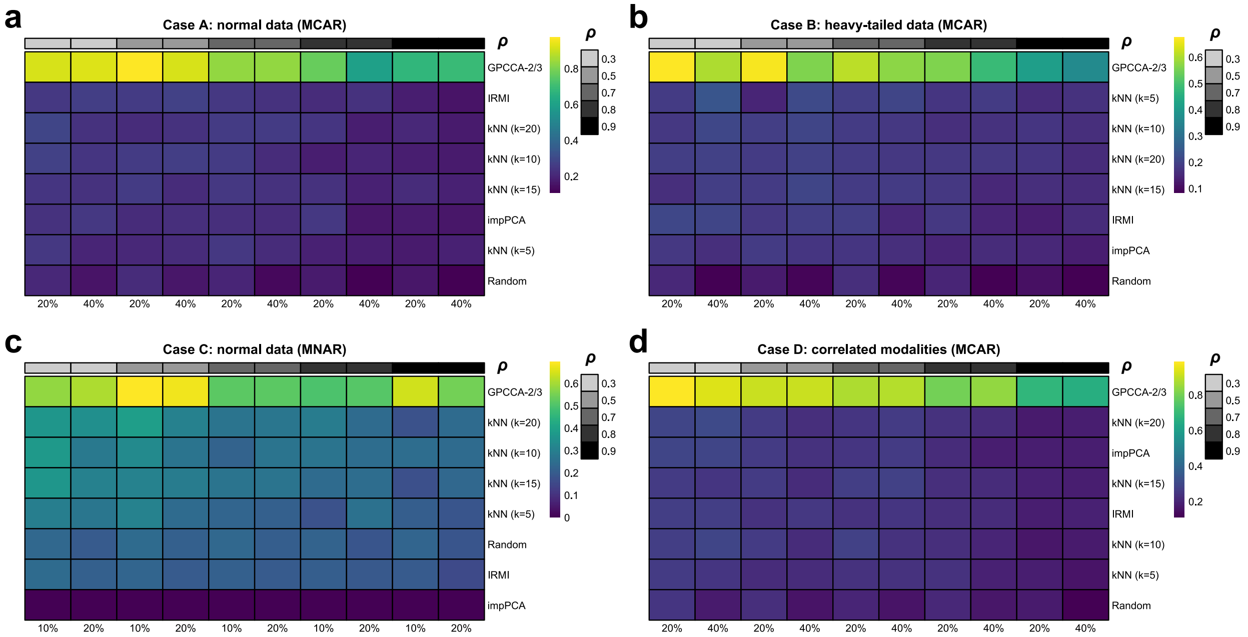

Figure S18: Comparison of clustering performance (ARI) of SNF on simulated datasets with different missing imputation techniques. **a-d** correspond to the four cases in our simulation study. Methods are ordered from highest to lowest based on their average ARI across all scenarios with different missing levels and correlation levels.

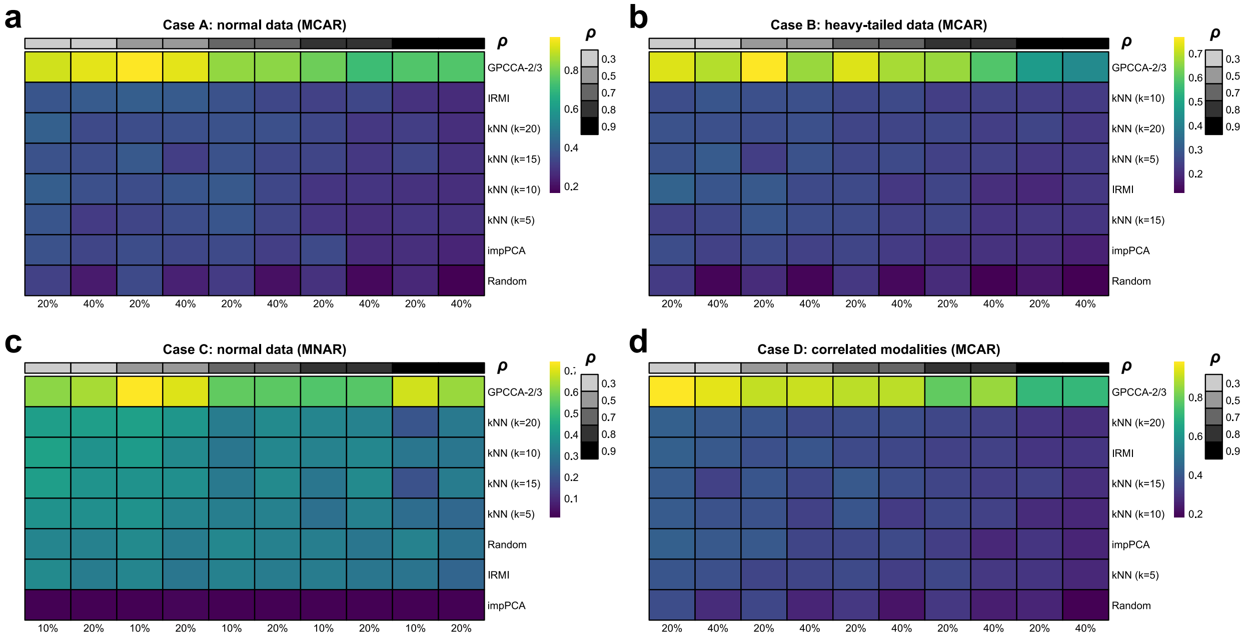

Figure S19: Comparison of clustering performance (NMI) of SNF on simulated datasets with different missing imputation techniques. **a-d** correspond to the four cases in our simulation study. Methods are ordered from highest to lowest based on their average NMI across all scenarios with different missing levels and correlation levels.

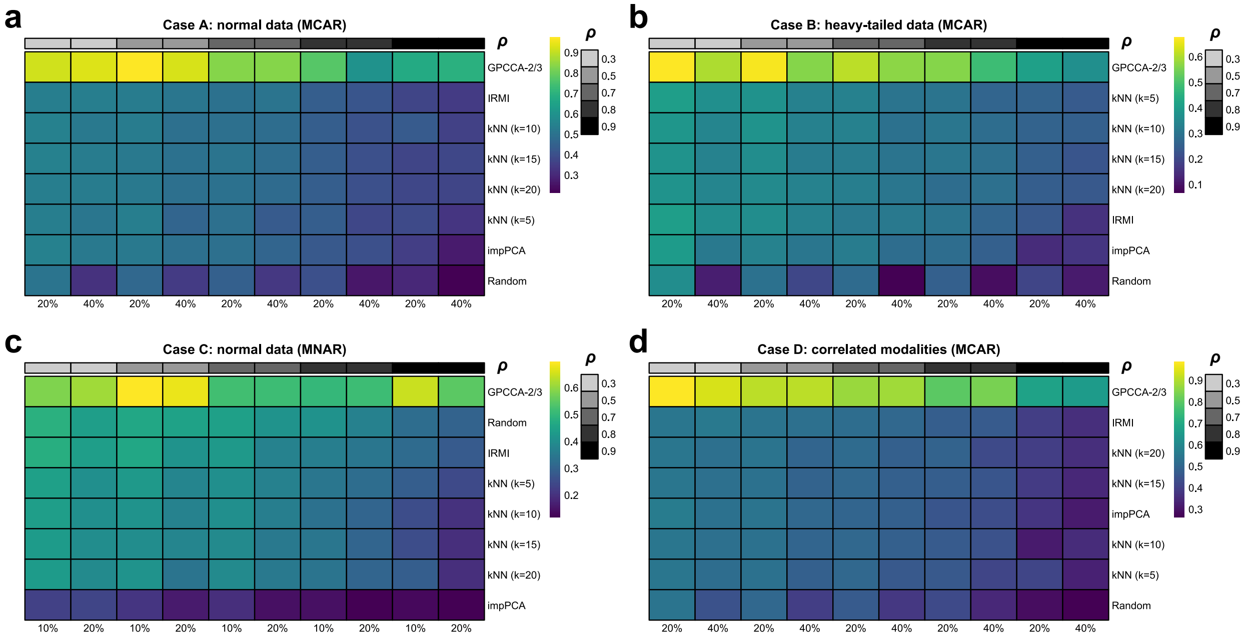

Figure S20: Comparison of clustering performance (ARI) of NEMO on simulated datasets with different missing imputation techniques. **a**-**d** correspond to the four cases in our simulation study. Methods are ordered from highest to lowest based on their average ARI across all scenarios with different missing levels and correlation levels.

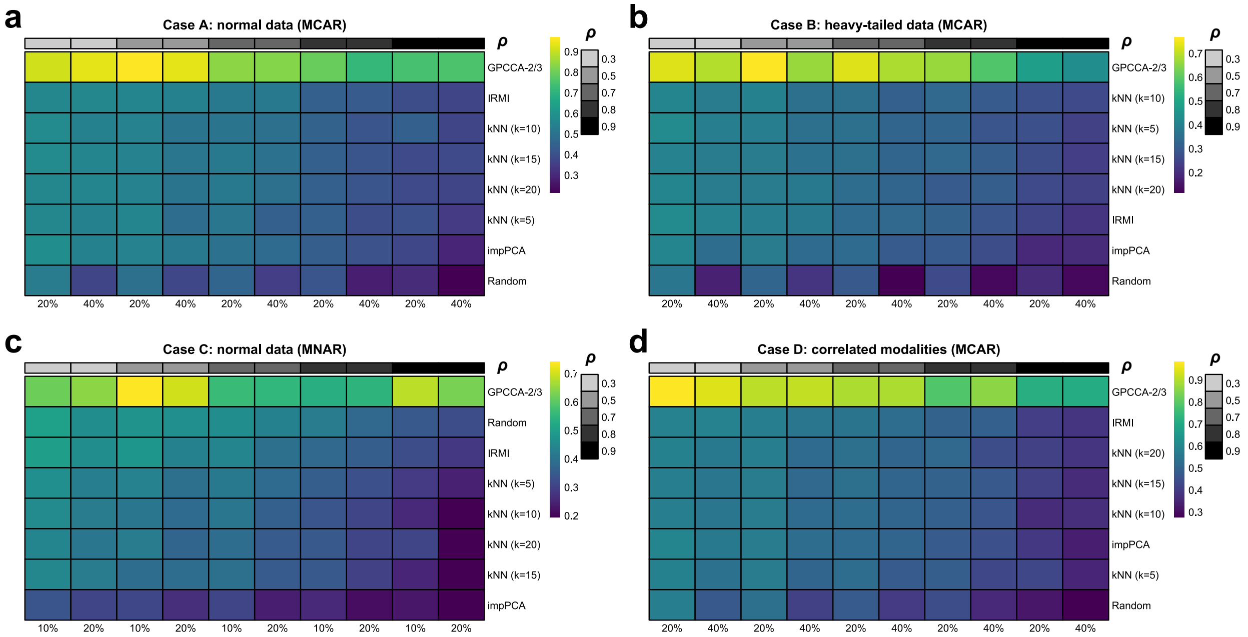

Figure S21: Comparison of clustering performance (NMI) of NEMO on simulated datasets with different missing imputation techniques. **a-d** correspond to the four cases in our simulation study. Methods are ordered from highest to lowest based on their average NMI across all scenarios with different missing levels and correlation levels.

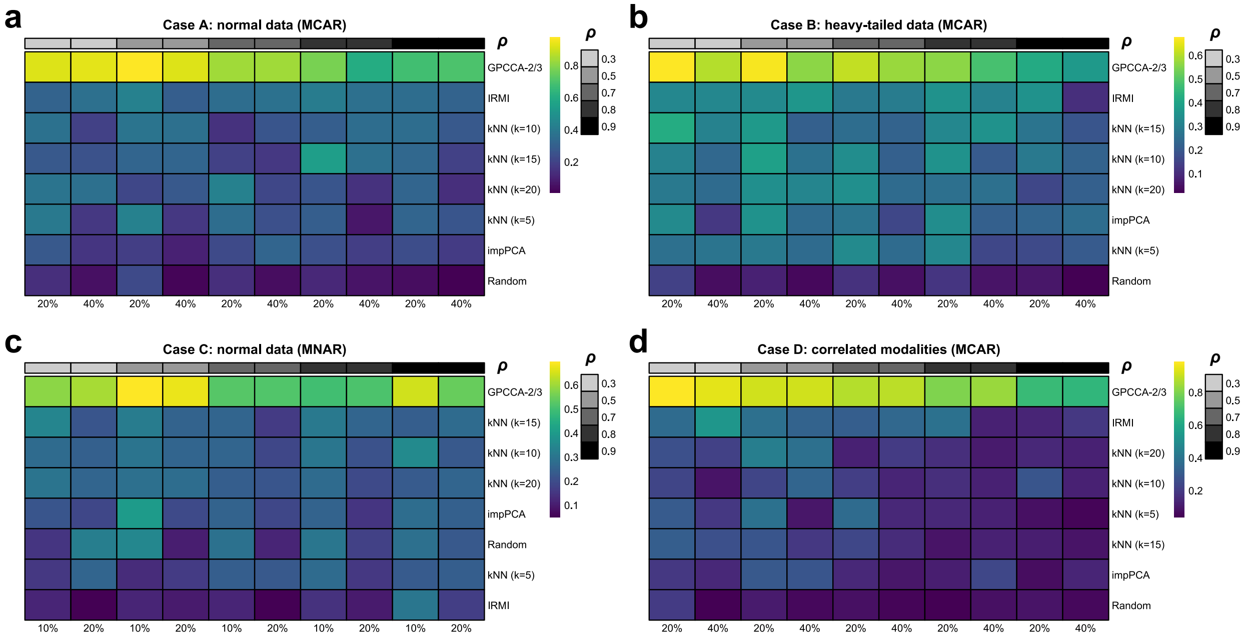

Figure S22: Comparison of clustering performance (ARI) of DGCCA on simulated datasets, with different missing imputation techniques. **a-d** correspond to the four cases in our simulation study. Methods are ordered from highest to lowest based on their average ARI across all scenarios with different missing levels and correlation levels.

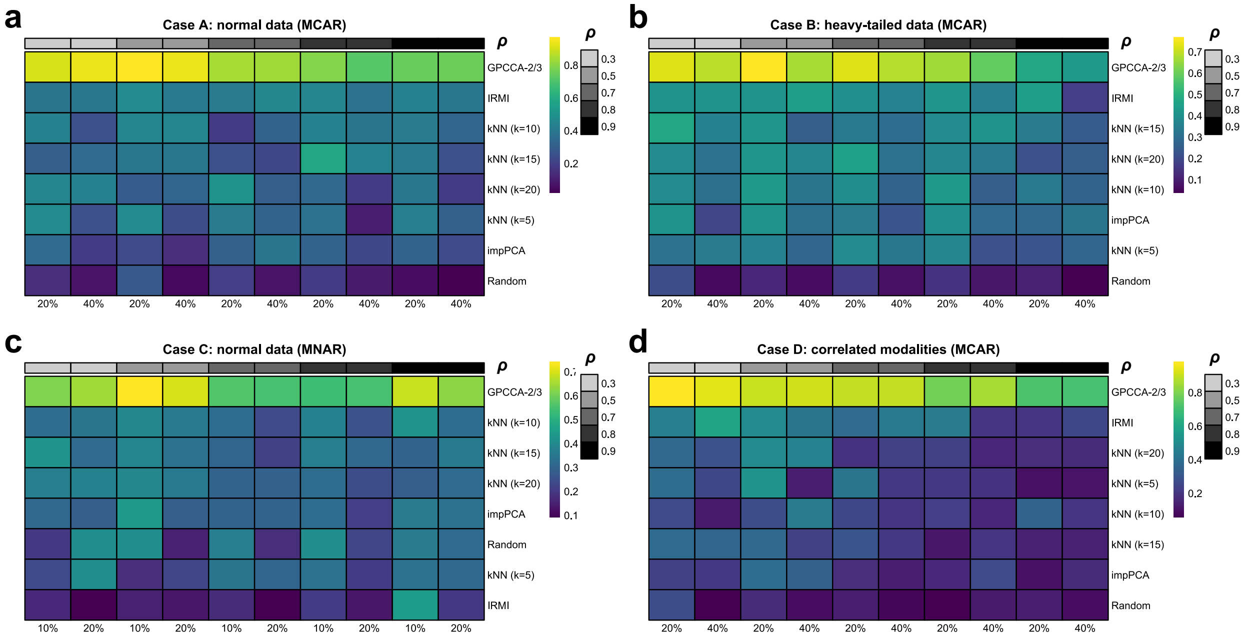

Figure S23: Comparison of clustering performance (NMI) of DGCCA on simulated datasets with different missing imputation techniques. **a**-**d** correspond to the four cases in our simulation study. Methods are ordered from highest to lowest based on their average NMI across all scenarios with different missing levels and correlation levels.

References

[1] Zoubin Ghahramani, Geoffrey E Hinton, et al. The em algorithm for mixtures of factor analyzers. Technical report, Technical Report CRG-TR-96-1, University of Toronto, 1996.

[2] N Benjamin Erichson, Sergey Voronin, Steven L Brunton, and J Nathan Kutz. Randomized matrix decompositions using r. *arXiv preprint arXiv:1608.02148*, 2016.

[3] H Thomas et al. Introduction to algorithms, 2009.

[4] David I Warton. Penalized normal likelihood and ridge regularization of correlation and covariance matrices. *Journal of the American Statistical Association*, 103(481):340–349, 2008.
